# Characteristics of Abnormalities in Somatosensory Submodalities Observed in Residents Exposed to Methylmercury

**DOI:** 10.3390/toxics11121023

**Published:** 2023-12-15

**Authors:** Shigeru Takaoka, Tadashi Fujino, Shin-ichi Shigeoka, Takashi Yorifuji

**Affiliations:** 1 Kyoritsu Neurology and Rehabilitation Clinic, 2-2-28 Sakurai-cho, Minamata 867-0045, Japan; 2 Minamata Kyoritsu Hospital, 2-2-12 Sakurai-cho, Minamata 867-0045, Japan; tds-fujino@jcom.zaq.ne.jp (T.F.); shigeoka@mk-kyouritu.com (S.-i.S.); 3 Department of Human Ecology, Graduate School of Environmental and Life Science, Okayama University, 3-1-1 Tsushima-naka, Kita-ku, Okayama 700-8530, Japan; yorichan@md.okayama-u.ac.jp

**Keywords:** methylmercury, somatosensory disturbance, somatosensory submodalities, somatosensory quantification, nerve conduction study

## Abstract

Hundreds of thousands of people living along the Yatsushiro Sea coast have been exposed to methylmercury from the contaminated water of the Chisso factory in Minamata. The most common neurological disorder caused by methylmercury is somatosensory disturbance, but very few studies have been conducted in the world to determine its pathophysiology and origin, including the Japanese cases, which have produced numerous intoxicated individuals. We have already shown in previous studies the body part where the disorder occurs and that its cause is not peripheral nerve damage but damage to the parietal lobes of the cerebrum. We reanalyzed the results of subjective symptoms, neurological findings, and quantitative sensory measurements in 197 residents (63.2 ± 10.7 years old) from contaminated areas exposed to methylmercury from seafood and 130 residents (63.7 ± 9.3 years old) from control areas, the same subjects as in previous studies, to determine the characteristics of somatosensory disturbance in detail. The most commonly affected sensory modalities were superficial peripheral touch and pain in the extremities, followed by two-point discrimination and deep senses, and in the most severe cases, full-body sensory dysfunction and impairment of all sensory submodalities. The severity of sensory submodalities correlated with each other but not with peripheral nerve conduction test indices, further confirming the correctness of our assertion about the responsible foci of sensory disturbance. The health effects of chronic methylmercury toxicosis can be elucidated by a detailed examination of sensory deficits.

## 1. Introduction

Methylmercury damages neurons in the cerebral cortex and cerebellar cortex, causing significant encephalopathy such as convulsions and impaired consciousness at high concentrations [1,2,3,4]. In severe cases, neurological disorders known as Hunter–Russell syndrome occur, including visual field constriction, hearing impairment, ataxia, and sensory disturbances. In mild cases, only somatosensory neuropathy may be observed in some cases. In milder cases, psychomotor deficits are known to occur at levels that do not cause sensory disturbances, particularly in fetal and pediatric exposures [5,6,7].

Methylmercury toxicosis in Minamata, Japan and Minamata disease in Niigata, Japan are characterized by an outbreak of toxicosis with motor and sensory disturbances that can be individually diagnosed with high epidemiologic probability.

Minamata disease was caused by the consumption of seafood contaminated with methylmercury [3]. Chisso Corporation in Minamata used mercury as a catalyst in the production of acetaldehyde from 1932 to 1968, and discharged mercury- and methylmercury-contaminated waste into Minamata Bay and the Yatsushiro Sea. Approximately 500,000 people lived in the region; until the 1960s, Japan was not fully economically developed, and the main source of protein for the region’s residents was fish and shellfish. This suggests that there was probably an exposed population of several hundred thousand people.

When Minamata disease occurred in 1956, toxicology was underdeveloped in Japan and the importance of epidemiological methods was not fully recognized. When the second Minamata disease occurred in Niigata in 1965, Dr. Tadao Tsubaki, one of the founders of the Japanese Society of Neurology and the first professor of the Department of Neurology at Niigata University, who was considered an authority on Minamata disease, conducted epidemiological research in the contaminated area following the teachings of Dr. Kurland [8,9].

However, in 1974, without referring to any patient data, he denied the diagnostic significance of sensory disturbance in methylmercury toxicosis and claimed that it was difficult to diagnose Minamata disease [10]. This was the beginning of a dark period for Minamata disease research in Japan. At the same time, there were almost no detailed studies on the sensory disturbance in Minamata disease.

Under these circumstances, some medical doctors began research on sensory disturbance in Minamata disease in the late 1990s. Through sensory quantification of patients with certified Minamata disease and those exposed to methylmercury, Dr. Ninomiya, Dr. Ekino, and colleagues found that the parietal cortex of the cerebrum was the responsible lesion for sensory disturbance in Minamata disease, and that the parietal cortex was also responsible for peripheral sensory disturbance in the limbs [11,12].

We started our research on the sensory disturbance around 1999, using the method of Ninomiya et al. and published our previous study in 2008 [13]. In this previous study, when the exposed groups were compared with groups with and without neurologically related complications, slightly more of those with complications showed abnormalities in complaints, neurological examination, and quantitative sensory measurements, compared with those without complications. However, the differences were very small compared with those in the controls, and it was concluded that most of the sensory deficits in these exposed individuals were due to methylmercury exposure, and that the complications had little effect.

Somatosensory submodalities can be divided into superficial (e.g., touch and pain from standard examination, minimal tactile and vibration), deep (e.g., position), and cortical (e.g., two-point discrimination) senses. Somatosensory dysfunction due to methylmercury toxicosis may involve all submodalities in severe cases, but fewer submodalities are affected in milder cases. There are reports that the superficial senses are more likely to be impaired than the positional senses, [14] but very few studies have examined such submodalities in detail.

In the previous study, the frequency of abnormalities in each submodality was calculated between the exposed and control groups. However, we did not analyze how the individual subjects differed in the manner in which they were impaired in touch and pain senses as the standard neurological examination, or in minimal tactile, vibration, position, and two-point discrimination senses as quantitative measurements; nor did we analyze what the relationships between these submodalities are.

In the present study, we aimed to provide a broader picture of the nature of sensory disturbance in methylmercury toxicosis by analyzing in detail the relationship between these individual subjective complaints, touch and pain senses in the standard medical examination, and quantitative sensory measurements. In particular, we thought it would be possible to clarify the relationship between peripheral sensory disturbance of the extremities and generalized sensory disturbance that can occur in methylmercury toxicosis, which has not been clarified in previous studies.

In the previous study, the relationship between subjective complaints expressing activities of daily living (ADLs) and sensory disturbance upon examination was not analyzed in individual subjects, but by clarifying this relationship, we thought it would be possible to learn how health problems manifest themselves in people exposed to methylmercury and to estimate the extent of health problems. In particular, we thought it would be possible to clarify the difference in etiology and pathophysiology between generalized sensory disturbance caused by methylmercury toxicosis and that caused by rare peripheral neuropathies.

In general, peripheral neuropathy is divided into axonal and myelin sheath (Schwann cell) damage, with myelin sheath damage resulting in decreased nerve conduction velocity and axonal damage resulting in decreased nerve conduction potential (amplitude). The previous study showed no correlation between sensory nerve conduction velocity and sensory quantification, indicating that the myelin sheath of peripheral nerves was not affected by sensory disturbance due to methylmercury toxicosis, supporting the assertion of Ninomiya et al. However, because the results of peripheral nerve amplitude were not reported, the presence or absence of axonal peripheral neuropathy was not adequately investigated and analyzed in the present study.

## 2. Materials and Methods

### 2.1. Subjects

The subjects of the exposed group in this study were the same 197 residents (63.2 ± 10.6 years, 37–89 years) who had lived in methylmercury-contaminated areas, consumed seafood, and had their health examined, as in our previous study published in 2008 [13]. Then, because the 227 subjects in the control area included many young people (53.0 ± 14.9 years, 30–86 years), 130 of these residents aged 50 years or older (63.7 ± 9.3 years, 50–86 years) were used as the control group.

In the previous study, residents in the control area who could have neurologically related complications were excluded from the control area, but the effect of complications was considered to be so small that 10 residents who had such complications (five with various types of cancer, one with cerebral haemorrhage sequelae, one with diabetes, one with lumbar spondylosis, one with cervical spondylosis, and one with cubital tunnel syndrome), were not excluded in the present analysis.

### 2.2. Epidemiologic Conditions and Questionnaire on Complaints

The questionnaire was the same as that used in previous studies. It included information to estimate methylmercury exposure, such as the subject’s residence, dietary habits, and occupational history, as well as medical history and the health status and history of family members. For subjective complaints, 50 questions were asked about sensory disturbances, motor disturbances, body aches, general complaints, and psychological and intellectual problems, and rates of usual and occasional symptoms were calculated. The subjects were asked to complete the questionnaire before the examination, but those who were unable to do so were interviewed.

### 2.3. Standard Neurological Examination

All subjects underwent a standard neurological examination. The results of dysarthria, hearing impairment, visual field constriction, finger-nose test, diadochokinesis, heel-shin test, gait disturbance, tandem gait, Romberg’s sign, standing on one leg with eyes open, and superficial sensory disturbance (touch and pain) were determined.

Dysarthria, hearing impairment, and visual field were assessed by the examiner without the use of special equipment. Visual field impairment was considered present if the confrontation method showed a lateral visual field of less than 80 degrees. Limb and truncal ataxia were scored as absent (−), mildly abnormal (+), or moderately or severely abnormal (++). Finger-nose and heel-shin tests were scored ++ if there was consistent measurement impairment or motor decomposition, and + if there was inconsistent measurement impairment, motor decomposition, or delayed reaching. Dysdiadochokinesis was scored ++ if there were consistent abnormalities and + if there were inconsistent abnormalities or slow movement. Tandem gait disorder was scored ++ if the subject could not take more than five steps, and + if the subject could take five steps but was unstable. One-foot standing was ++ if the subject could not stand with eyes open for more than three seconds, and + if the subject could stand with eyes open for more than three seconds. In our study, the percentages of ++ and + were summed.

Touch disturbance was tested by a light touch with a brush, comparing the periphery of the upper and lower limbs with the chest or the central part of the upper and lower limbs. Touch disturbance in the chest or trunk was determined by whether the person could feel a light touch with the tip of a brush, and sometimes the response of whether the person could feel a light stroke with tissue paper was taken into account. Pain disorder was tested with a pain needle, and the disorder was considered present if the pain was not felt or was felt weakly. Pain disorder in the chest and trunk was assessed by the pain response to the pain needle. In the present study, we also calculated the frequency of perioral touch and pain disturbance, which had not been analyzed in previous studies.

All the physicians participating in the study were trained by document, direct instruction, or videotape. The neurological examinations were performed in two phases. The first phase was performed by seven physicians in the exposed group and 49 physicians in the control area. In the second phase, in both the contaminated and control areas, checking for superficial sensory disturbance and quantitative sensory examination for minimal tactile and two-point discrimination senses was performed by two physicians of Minamata Hospital (S.T. and Y.K.).

**Figure 1:**
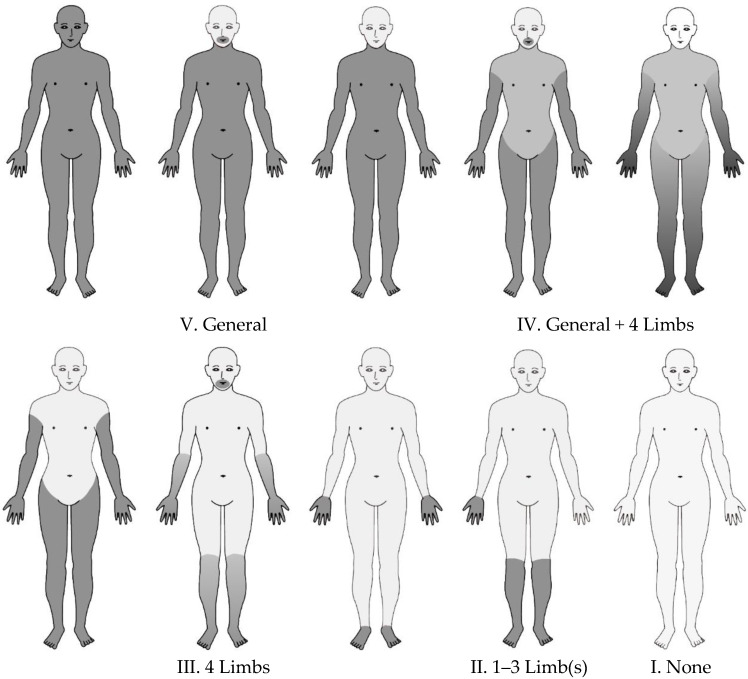
Examples of somatosensory disturbance types due to methylmercury exposure.

The results of the standard superficial touch and pain sensory examination of each group were classified into five categories (Figure 1): V. equally impaired generalized (whole body, except for face and head) sensory disturbance; IV. generalized plus four-limb dominant sensory disturbance; III. four-limb dominant sensory disturbance without generalized sensory disturbance; II. sensory disturbance of one to three limbs, and; I. no limb sensory disturbance.

The above was the same as in previous studies, but in the present analysis, we graded the patients according to the severity of their superficial sensory disturbance (touch and pain) on the standard neurological examination (Table 1). Level 4 for generalized impairment of both touch and pain senses, level 3 for generalized impairment of either touch or pain senses, level 2 for four-limb dominant impairment of both touch and pain (with no generalized impairment of both touch and both), level 1 for four-limb dominant impairment of either touch or pain (with no generalized impairment of both touch and both), and level 0 for no four-limb dominant abnormalities of both touch and pain senses.

### 2.4. Quantitative Sensory Measurements

Vibration and position senses were measured by each physician. Minimal tactile and two-point discrimination senses were measured by two trained physicians from the Minamata Kyoritsu Hospital. The temperature of the laboratory was maintained between 23 and 27 degrees Celsius during the sensory measurements.

#### 2.4.1. Minimal Tactile Sense by Semmes–Weinstein Monofilaments

After completing the standard sensory examination, the minimal tactile sense was measured with Semmes–Weinstein monofilaments. We used 20 types of filaments ranging from 0.008 g to 300 g. Subjects were tested with their eyes closed after receiving clear instructions. Each filament was squeezed until it bent approximately 90 degrees for approximately one second. The threshold was the smallest filament size that a subject could feel as a touch. Each trial was performed once with each filament, except when the subject was unsure, in which case the examiner provided an odd number of trials with the same filament and selected the answer given in over 50% of the trials. If a subject could not detect the maximum filament (300 g), we defined the threshold as 400 g for calculation. Examination was performed on the lower lip, chest, and ventral sides of both index fingers and great toes.

We did not use the gram weight to calculate the minimum tactile sense, but instead converted it to logarithmic values using the following equation:

Evaluator size = log ([grams]) + 4.

#### 2.4.2. Vibration Sense

Vibration sense was measured by using a 128 Hz tuning fork. The examiner fully knocked the tuning fork and started the stopwatch at the same time. Subjects were instructed to report immediately when they could no longer feel the vibration at all, and the time up to that point was recorded. Vibrations were measured at the upper sternum, the radial side of both wrists, and the fibular side of both ankles.

#### 2.4.3. Position Sense

Position sense was measured with the subject’s eyes closed using a ruler with millimeter lines. Each examiner held the lateral side of the finger or toe and moved it up or down for approximately one second, using the horizontal position on the outside of the nail as the zero point. The minimum distance at which the subject could perceive the direction was 5 mm, and the examiner increased the distance by 5 mm up or down. Each trial was a single trial, but if the subject’s response was ambiguous, the examiner checked an odd number of times and selected the one that was answered more than 50% of the time. If the subject could not feel the position with maximum movement, the threshold was defined as the maximum plus 5 mm. An examination was performed with the index finger and the great toe.

For one subject in the exposed group, thresholds were entered on the recording form at a distance other than the predetermined distance (2, 4, 6 mm) in the bilateral upper and bilateral lower directions, but these values were used in the calculation without modification because they were considered to have little effect.

#### 2.4.4. Two-Point Discrimination Sense

Two-point discrimination threshold was determined with the subject’s eyes closed using a drafting divider. The divider was placed on the skin at an angle of 30–45 degrees and a depth of 1–2 mm for approximately one second. A two-alternative, forced-choice technique was used. The distances tested were 1, 2, 3, 4, 5, 6, 8, 10, 12, 15, 20, 25, 30, and 36 mm. The threshold was defined as the lowest distance at which a subject responded correctly on all three consecutive trials. The starting point distance was estimated by each clinician after observing the overall state of sensory impairment in order to reduce the testing time and avoid fatigue. This method was performed on the lower lip and ventral side of each index finger. If a subject could not respond to the maximum distance (36 mm), the threshold was defined as 40 mm for calculation.

In the exposed groups, the threshold distances differed from the predetermined distances (7, 9, 14, 16, 18, and 26 mm) on the recording form for two subjects at the lower lip, two subjects at the right index finger, and four subjects at the left index finger. Since the influence was judged to be small, these values were used in the calculations as they were, without modification.

### 2.5. Neurophysiologic, Neuroradiological, and Other Laboratory Tests

Neurophysiologic examination was performed only in the exposed group and included Goldmann’s perimeter, audiometry, and nerve conduction studies. Neuroradiological examination included cervical spine radiographs (6 views), lumbar spine radiographs (4 views), and head CT. Biochemical tests included blood glucose, HTLV-I antibodies, and LE test or antinuclear antibodies (FA method). These tests were not performed in the control subjects.

In the previous study, we used only sensory and motor nerve conduction velocities of the median nerve for comparison with sensory measurements, whereas in the present study, we used the results of sensory nerve conduction velocities and amplitudes of the median and peroneal nerves. Sensory nerve conduction velocities were measured by retrograde methods. Detailed methods were as described in previous studies.

Neurophysiologic studies were performed in 187 of 197 subjects in the exposed group. Conduction velocity data had been recorded in a previous study, but potential data had not been compiled, so the original data were used again in this study. A number of 14 of the 187 subjects had lost their original data, so potential data were used in 173 subjects. However, since no bias factor was found in the lost data, it is considered that there is no statistical problem.

### 2.6. Statistical Methods

In the previous study, we used the presence of other neurologically related disorders to divide the exposed subjects into groups with complications (E + N) and without complications (E). One hundred and seventeen subjects had at least one complication. The complications were as follows: diabetes mellitus (34), cervical spondylosis (54), lumbar radiculopathy (15), carpal tunnel disease (42), cerebrovascular disease (29), and other diseases (14). The criteria for each were shown in previous studies and were based on the tests introduced in 2.5. On the other hand, complications in the control group were based only on the medical interview.

Other neurologic abnormalities included chronic psychiatric drug users (3), mental retardation (3), hypothyroidism (3), cubital tunnel syndrome (2), other polyneuropathies (2), HTLV-I-associated myelopathy (1), spinocerebellar degeneration (1), and epilepsy (1).

In the previous study, the exposed group was divided into two groups, E and E + N, according to strict criteria, including several tests, to analyze the effects of neurologically related diseases on neurological signs. However, among the neurologically related diseases listed in the exposure groups, those that primarily cause neurological symptoms similar to those of methylmercury toxicosis are rare, and none of them causes generalized sensory disturbances. We believe that the much stricter criteria for complications in the exposed group than in the control group is not necessarily a positive bias.

Diseases that can cause peripheral sensory disturbances in the extremities include polyneuropathies such as diabetic peripheral neuropathy. However, in diabetic polyneuropathy, sensory disturbances are often present in the lower extremities and absent or weak in the upper extremities, whereas in methylmercury toxicosis, sensory disturbances are often present in the upper and lower extremities to the same degree. The peripheral sensory disturbances in the extremities of the subjects in the present study, which will be described later, were of the latter type.

In the previous study, the percentages of generalized touch disturbance in the control, E, and E + N groups were 0%, 22%, and 14%, respectively; generalized pain disturbance was 0%, 44%, and 44%, respectively; no touch disturbance was 97%, 9%, and 8%, respectively; no pain disturbance was 97%, 4%, and 3%, respectively.

The results, for example, of the quantitative sensory examination of the right hand were as follows. Threshold of minimal tactile sense (evaluator size) in the right index finger for the control, E, and E + N groups was 3.07 ± 0.39, 4.12 ± 0.57, and 4.38 ± 0.71, respectively; threshold of vibration sense (right wrist) was 17.0 ± 3.2 s, 10.0 ± 3.4 s, and 8.7 ± 3.7 s, respectively; threshold of two-point discrimination sense (right index finger) was 2.7 ± 1.2 mm, 14.9 ± 13.3 mm, and 18.3 ± 15.2 mm, respectively.

Compared to the E group, the E + N group showed worse sensory disturbances in all modalities, but these were much milder than the differences between the control and the respective groups. Therefore, we decided to compare the E and E + N groups together with the control group in the present study. We believe that the effect of complications, although undeniable, is not significant.

Statistical calculations were performed using MS-Excel and STATA software (version 14).

#### 2.6.1. Questionnaire and Neurological Examination

To analyze the questionnaire data, the percentages of “always” and “always or sometimes” responses were summed, and the results were compared among Group E, Group E + N, Exposed (total), and Control.

#### 2.6.2. Percentage of Touch and Pain Disturbance

The results of the standard examination for touch and pain disturbance of the exposed groups were compared in terms of the percentage of the five categories of disturbance (Figure 1V–I).

#### 2.6.3. Relationship between Complaints and Sensory Disturbance Level

We analyzed the frequency of subjective complaints according to the level of superficial sensory disturbance (Table 1, Level 4–0).

These included questions related to sensory disturbance (Q1, Q2, Q5, Q6, Q7), a question about movement (Q32), questions related to both sensory disturbance and motor stability disturbance (Q22, Q23, Q24, Q26, Q27, Q28), questions about pain (Q6, Q7), question about vision (Q12, Q Q14), question about smell (Q19), and a question about taste (Q20).

#### 2.6.4. Relationship between Touch Disturbance Category (V–I) and Results of Quantitative Sensory Measurements, Perimeter, and Audiometry

We compared the touch disturbance category (I–V) and the thresholds of minimal tactile, vibration, position, and two-point discrimination senses, as well as the results of perimeter and audiometry, to know the relationship between standard touch examination and quantitative test values, to analyze the relationship and continuity between generalized sensory disturbance and peripheral sensory disturbance of the extremities, and to analyze the relationship with visual and auditory abilities.

#### 2.6.5. Relationship Minimal Tactile Sense and Two-Point Discrimination Sense

We compared the correlations between minimal tactile sense and two-point discrimination sense in the exposed and control groups, respectively, in order to understand the relationship between superficial touch disturbance and cortical sensory disturbance.

#### 2.6.6. Relationship between Quantitative Sensory Measurements and Results of Evoked Electromyography

The relationship between minimal tactile, vibratory, positional, and two-point discrimination senses and the sensory nerve conduction velocity (SCV) and amplitude (SCA) of the median and peroneal nerves was examined. In this analysis, multiple regression analysis was used to determine whether there was a relationship between the results of each quantitative sensory measurement and SCV and SCA, adjusting for age, sex, and complications that could affect the nervous system.

Minimal tactile sense, position sense, and two-point discrimination sense of the bilateral index fingers, and the vibration sense of the bilateral wrists were compared with the SCV and SCA of the ipsilateral median nerve. Minimal tactile sense, position sense, and two-point discrimination sense of the bilateral great toes and the bilateral vibration sense of the bilateral ankle were compared with the SCV and SCA of the ipsilateral peroneal nerve.

## 3. Results

### 3.1. Backgrounds of Subjects

The background of the subjects is shown in Table 2. All of the exposed groups lived in methylmercury-contaminated areas and were exposed to methylmercury through their own and their family members’ occupational and seafood consumption histories. The percentage of persons who were fishermen or belonged to a family of fishermen was significantly higher in Group E (11.1% of the subjects, 45.7% of the families) and Group E + N (6.9%, 23.7%) than in the control group (0%, 1%). Seventy-two percent of the exposed group (total) had at least one family member who had received compensation for Minamata disease. The frequency of fish intake was also significantly higher in Group E and Group E + N than in the control group.

### 3.2. Complaints and Neurological Examination

In chronic methylmercury toxicosis, it is difficult to estimate the total exposure even when hair mercury levels are examined. In addition, methylmercury exposure has not been measured in residents of contaminated areas in Japan. Therefore, comparison of subjective complaints and neurological findings with a control group is helpful in estimating the overall health effects.

The results of the complaints questionnaire are shown in Table 3 and Table 4. These results are almost identical to those presented in the previous study. All complaints were significantly higher in the exposed group than in the control group. Five complaints with the answer “always” and three with the answer “always + sometimes” were more frequent in Group E + N than in Group E. This difference may be due to complications and age differences, but it was considerably smaller than the difference between the control group and the two groups.

The results of the neurological examination are shown in Table 5. All complaints were significantly higher in the exposed group than in the control group, with large and significant differences: Group E + N had a significantly higher rate than Group E in 7 of the 21 items, but there were no significant differences in the other items.

### 3.3. Comparison of Touch and Pain Disturbance

The mode of occurrence of touch and pain disturbance in the exposed group was investigated by the standard sensory examination (Table 6). When the severity of touch and pain senses was classified into five categories (V–I), generalized (V + IV) sensory disturbance was 18% for touch and 40% for pain, and the combined generalized and peripheral sensory disturbance (V + IV + III) was 83% for touch and 95% for pain, indicating that pain disturbance was more likely to be found than touch disturbance. The severity of sensory disturbance was often concordant, but not necessarily in the same category.

The generalized sensory disturbance was divided into cases in which the trunk and peripheral extremities were approximately equal (V) and those in which the peripheral extremities were more affected than the trunk (IV), and a comparison of the severity of the two groups was later analyzed by quantifying touch sense.

### 3.4. Relationship between Complaints and Sensory Disturbance Level (Level 4–0)

The relationship between the superficial touch and pain disturbance level and subjective complaints expressing each resident’s ADLs is shown in Figure S1 through Figure S18. Overall, it was found that the higher the level of sensory disturbance, the higher the rate of subjective complaints. However, some patients had no complaints even at the severe disturbance level and some had complaints at the mild disturbance level.

The prevalence of headache (Q8, Figure S6) and cramps (Q11, Figure S7) did not necessarily correlate with the sensory disturbance level. Complaints of vision (Q12, Figure S8), smell (Q19, Figure S10), and taste (Q20, Figure S11) tended to increase with the sensory disturbance level, but a certain percentage of people had complaints even at level 0.

These data suggest that somatosensory disturbance, as a whole, reflects the severity of the disturbance caused by methylmercury toxicosis. At the same time, they suggest that even if somatosensory disturbance is milder or even absent in some individuals, there may be mild abnormalities in other areas, such as vision, smell, taste, smooth motor movement, and balance.

### 3.5. Relationship between Touch Disturbance Categories (V–I) and Quantitative Sensory Measurements, Perimeter, and Audiometry

When examining the relationship between each touch disturbance category (V–I) and quantitative sensory measurements, there were significant age differences between the exposed group with 1–3 limb sensory abnormalities (II) and the control group (Table 7 and Table 8). The possible influence of age differences must be considered in the analysis of the following data, but the effect is not considered to be large.

**Figure 2:**
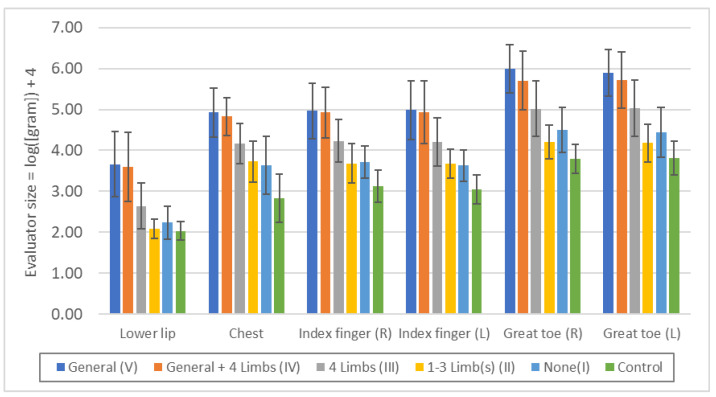
Threshold of “minimal tactile sense” in each touch disturbance type by examination. Lower lip: n.s.: V/IV, II/I, II/Control, *p* < 0.05: I/Control, *p* < 0.01: all others. Chest: n.s.: V/IV, II/I, *p* < 0.01: all others. Index finger (R): n.s.: V/IV, II/I, *p* < 0.01: all others. Index finger (L): n.s.: V/IV, II/I, *p* < 0.01: all others. Great toe (R): n.s.: V/IV, *p* < 0.05: II/I, *p* < 0.01: all others. Great toe (L): n.s.: V/IV, II/I, *p* < 0.01: all others.

Minimal tactile sense was more disturbed in the order V ≥ IV > III > II = I > Control, but there were no significant differences between V and IV or between II and I (Figure 2, Table S1). It is not surprising that groups V and IV with touch disturbances in the standard examination, had disturbances not only in the extremities but also in the lower lip and chest, but even those with peripheral sensory disturbances only in the extremities (III) had significant differences in the lower lip and chest. Even those with sensory disturbances in 1–3 limb(s) (II) or no sensory disturbances (I) were also significantly different in the chest compared to controls. These indicate that in sensory disturbance due to methylmercury toxicosis, potential sensory disturbance also exists in the head face and chest.

**Figure 3:**
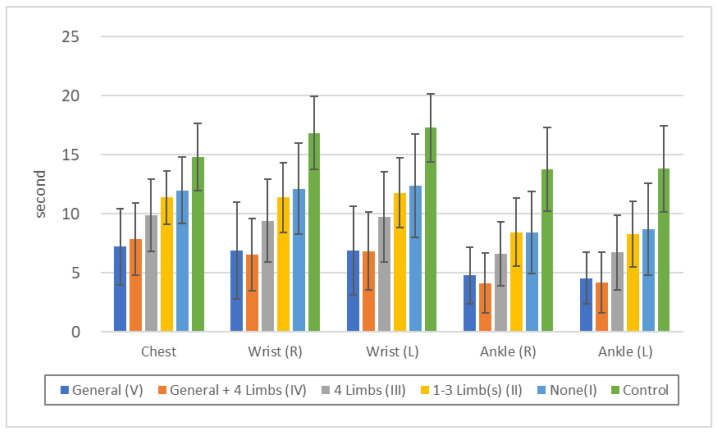
Threshold of “vibration sense” in each touch disturbance type by examination. Chest: n.s.: V/IV, II/I, *p* < 0.01: all others. Wrist (R): n.s.: V/IV, II/I, *p* < 0.05: V/III, *p* < 0.01: all others. Wrist (L): n.s.: V/IV, II/I, *p* < 0.05: III/I, *p* < 0.01: all others. Ankle (R): n.s.: V/IV, II/I, *p* < 0.05: III/I, *p* < 0.01: all others. Ankle (L): n.s.: V/IV, II/I, *p* < 0.05: III/II, III/II, *p* < 0.01: all others.

Vibration sense was more disturbed in the order V = IV > III > II > II ≥ I > Control, but there were no significant differences between V and IV or between II and I (Figure 3, Table S2). It is not surprising that the group V and IV with touch disturbance in the standard examination had disturbance in the chest as well as in the extremities, but those with sensory disturbance only in the peripheral extremities (III), those with sensory disturbance in one to three limbs (II), or no sensory disturbance (I) also showed significant differences in the chest compared to the controls. This indicates that in sensory disturbances due to methylmercury toxicosis, potential sensory disturbance also exists in the trunk.

**Figure 4:**
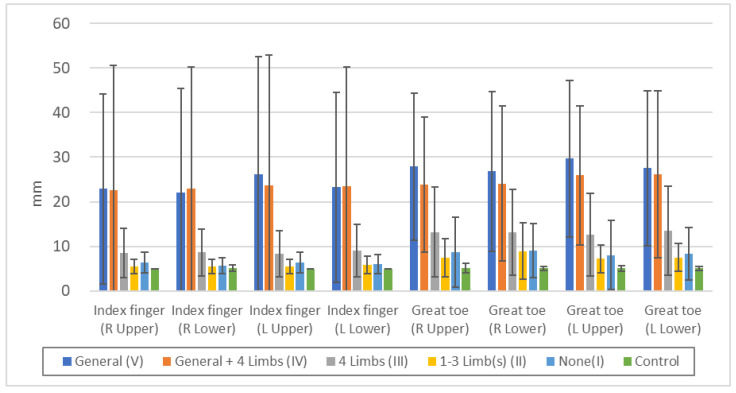
Threshold of “position sense” in each touch disturbance type by examination. Index finger (R Upper): n.s.: V/IV, II/I, II/Control, *p* < 0.05: IV/III, *p* < 0.01: all others. Index finger (R Lower): n.s.: V/IV, II/I, II/Control, I/Control, *p* < 0.05: V/III, *p* < 0.01: all others. Index finger (L Upper): n.s.: V/IV, II/I, II/Control, *p* < 0.01: all others. Index finger (L Lower): n.s.: V/IV, II/I, *p* < 0.05: II/Control, I/Control, *p* < 0.01: all others. Great toe (R Upper): n.s.: V/IV, II/I, *p* < 0.05: III/I, II/Control, I/Control, *p* < 0.01: all others. Great toe (R Lower): n.s.: V/IV, II/I, *p* < 0.05: III/I, *p* < 0.01: all others. Great toe (L Upper): n.s.: V/IV, II/I, I/Control, *p* < 0.05: III/I, *p* < 0.01: all others. Great toe (L Lower): n.s.: V/IV, II/I, *p* < 0.05: I/Control, *p* < 0.01: all others.

Position sense was more disturbed in the order V = IV > III > II > II = I > Control, but there were no significant differences between V and IV or between II and I (Figure 4, Table S3). Not a few of the subjects with peripheral sensory disturbance only in the extremities (III) are unable to detect abnormalities, suggesting that position sense disturbance is less likely to occur or be detected than superficial sensory deficits such as pain and touch.

**Figure 5:**
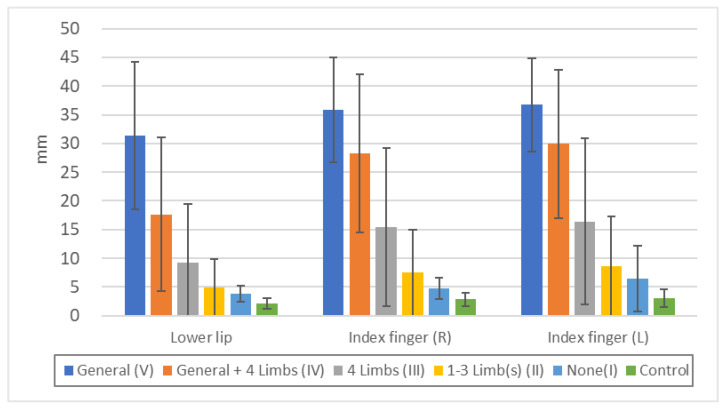
Threshold of “two-point discrimination sense” in each touch disturbance type by examination. Lower lip: n.s.: II/I, *p* < 0.01: all others. Index finger (R): *p* < 0.05: V/IV, II/I, *p* < 0.01: all others. Index finger (L): n.s.: II/I, *p* < 0.05: V/IV, I/Control, *p* < 0.01: all others.

Two-point discrimination was more disturbed in the order V > IV > III > II > I > Control, but there was no significant difference between II and I (Figure 5, Table S4). It is not surprising that the group V and IV with touch disturbance in the standard examination had disturbance in the chest as well as in the extremities, but those with sensory disturbance only in the peripheral extremities (III), those with sensory disturbance in one to three limbs (II), or no sensory disturbance (I) also showed a significant difference in the lower lip compared to controls. This indicates that in sensory disturbances due to methylmercury toxicosis, there are latent sensory deficits in various part of the body.

**Figure 6:**
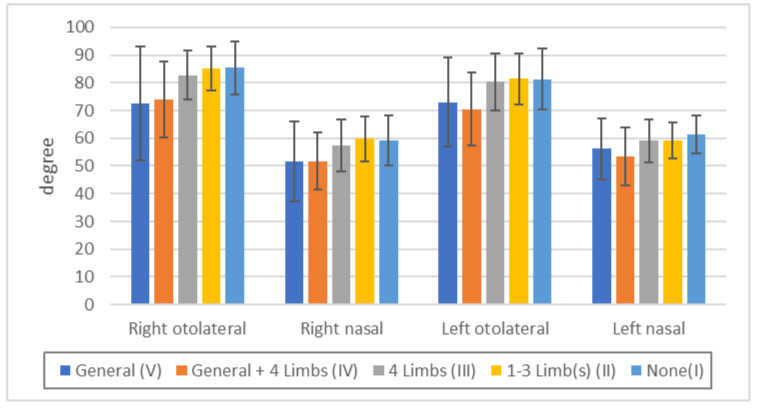
Threshold of visual field by Goldmann’s perimeter in each touch disturbance type by examination. Right otolateral: *p* < 0.01: IV/III, IV/II, IV/I, *p* < 0.05: V/III, V/II, V/I, n.s.: all others. Right nasal: *p* < 0.01: IV/III, IV/II, *p* < 0.05: V/III, IV/I, n.s.: all others. Left otolateral: *p* < 0.01: *p* < 0.01: IV/III, IV/II, IV/I, n.s.: all others. Left nasal: *p* < 0.01: IV/I, *p* < 0.05: IV/III, IV/II, n.s.: all others.

Visual field was more disturbed in the order V = IV > III > II = I, but there were no significant differences between V and IV, between III and I, or between II and I (Figure 6, Table S5). These data indicate that the intensity of visual field constriction parallels the severity of sensory disturbance and that sensory disturbance is a more sensitive indicator.

**Figure 7:**
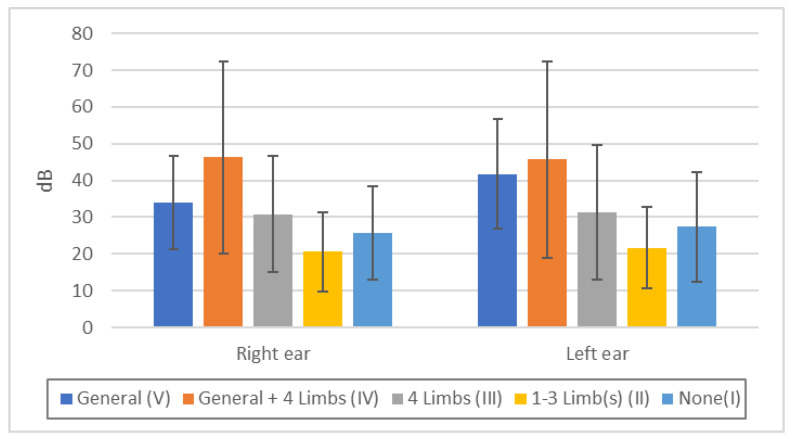
Auditory acuity by audiometer in each touch disturbance type by examination. Right ear: n.s.: V/III, III/I, II/I, *p* < 0.05: V/IV, V/I, *p* < 0.01: all others. Left ear: n.s.: V/IV, III/I, II/I, *p* < 0.01: all others.

Hearing was more disturbed in the order IV > V > III > I > II, but there was variability (Figure 7, Table S6). These data indicate that the intensity of hearing impairment parallels the severity of sensory impairment, but the correlation is smaller.

### 3.6. Relationship between Minimal Tactile Sense and Two-Point Discrimination Sense

The thresholds for minimal tactile sense and two-point discrimination sense were limited to a very narrow range in the control group, and the correlation was not always clear (Figures S20, S22 and S24). In contrast, in the exposed group, they all had a wide range, from mild to severe abnormalities, with large interindividual differences (Figures S19, S21 and S23).

This means that in the case of methylmercury poisoning, both the minimal tactile sense and the two-point discrimination sense are disturbed by the thinning-out mechanism of cortical neurons, but it is difficult to say which is more disturbed because there are individual differences in which is more disturbed.

### 3.7. Relationship between Quantitative Sensory Measurements and Results of Evoked Electromyography

We evaluated the relationship between quantitative sensory measurements and the results of evoked electromyography, i.e., sensory nerve conduction velocity (SCV) and amplitude (SCA), using multiple regression analysis that adjusted for age, sex, and neurologically related complications. Minimal tactile sense, position sense, and two-point discrimination sense of the bilateral index fingers, and the vibration sense of the bilateral wrists, were compared with the SCV and SCA of the ipsilateral median nerve. Minimal tactile sense and position sense of the bilateral great toes and the bilateral vibration sense of the bilateral ankle were compared with the SCV and SCA of the ipsilateral peroneal nerve.

Those results are shown in Supplementary Figures (Figure S25 through Figure S32, Figure S33 through Figure S40, Figure S41 through Figure S56, and Figure S57 through Figure S60) and Supplementary Tables (Table S7 through Table S14, Table S15 through Table S22, Table S23 through Table S48, Table S49 through Table S52).

There was no correlation at all between minimal tactile sense of the bilateral index fingers and the SCV and SCA of the ipsilateral median nerve (Figure S25 through Figure S28, Table S7 through Table S10), or between minimal tactile sense of the bilateral great toes and SCV and SCA of the ipsilateral peroneal nerve (Figure S29 through Figure S32, Table S11 through Table S14).

There was no correlation at all between bilateral wrist vibration sense and SCV and SCA of the ipsilateral median nerve (Figure S33 through Figure S36, Table S15 through Table S18), or between bilateral ankle vibration sense and SCV and SCA of the ipsilateral peroneal nerve (Figure S37 through Figure S40, Table S19 through Table S22).

There was no correlation at all between the position sense of the bilateral index fingers and SCV and SCA of the ipsilateral median nerve (Figure S41 through Figure S48, Table S23 through Table S30), or between the position sense of the bilateral great toes and SCV and SCA of the ipsilateral peroneal nerve (Figure S49 through Figure S56, Table S31 through Table S38).

There was no correlation at all between the two-point discrimination sense of the bilateral index fingers and SCV and SCA of the ipsilateral median nerve (Figure S57 through Figure S60, Table S39 through Table S42).

In addition to the previous studies showing no correlation between each quantitative sensory measurements and SCV, this study also found no correlation at all with SCA.

## 4. Discussion

It is well known that somatosensory disturbances occur when methylmercury toxicosis manifests; of the two deaths from the world’s first methylmercury toxicosis reported by Edwards, one patient reportedly complained of numbness in the hands and sensory dullness in the hands, feet, and tongue [1]. Hunter et al. reported four cases; case 1 was aware of generalized sensory numbness and tingling; all four cases had impairment of two-point discrimination, three had impairment of stereognosis, one had impairment of vibration, and there was no significant abnormality in touch or pain sense [2].

In the first report from Minamata, most cases showed subjective complaints of numbness in the limbs and mouth as well as sensory disturbance, and in the most severe cases there was generalized pain disturbance [15].

In the Iraqi cases, the initial symptoms were sensory disturbances in the extremities and perioral area. In a report of 19 patients examined by Le Quesne et al., four months after the onset of the disease, many patients had decreased pain, position, two-point discrimination, and stereognosis, and five patients had superficial sensory disturbances in the extremities and around and in the mouth, but no abnormal vibration sense [16].

Snyder et al. reported an acute severe case in which touch, pain, and vibration senses were normal and position sense, two-point discrimination sense, and stereognosis were disturbed [17], but they did not describe the details of their detection technique.

In a report at these lower concentrations of methylmercury in the Amazon, Oliveira et al. found abnormalities in distal pinprick perception, distal thermal sensitivity, hallux or thumb vibration sensitivity, feet mechanical detection [18], and Khoury et al. found abnormalities in microtactile perception, vibration sensitivity, and two-point discrimination, but reported that the degree and frequency of abnormalities were much lower than ours [19].

Many factors are thought to influence the degree and nature of sensory impairment: whether exposure to methylmercury is short term or long term, high dose or low dose, immediate or delayed exposure, and the specific method and criteria used to detect sensory disturbance may affect the degree and submodalities of the sensory disturbance detected, and the body site where sensory impairment occurs.

In addition, there is the additional issue of the relationship between exposure and symptom emergence. Nierenberg et al. reported a case of accidental exposure to dimethylmercury in which symptoms did not appear at the time of the highest hair mercury level of about 1000 μg/g (about 40 days after exposure), which should have remained parallel to blood mercury after exposure, but symptoms developed 154 days later, progressed to a fulminant form as methylmercury levels fell, and death occurred 298 days later [20]. This implies that there is a time lag between measurable blood levels and health problems (brain dysfunction) even in acute exposure. It should also be noted that it is difficult to know the exact dose–response relationship in methylmercury poisoning, considering that a single hair mercury value does not necessarily indicate an accurate total methylmercury exposure.

The variety and complexity of these symptoms may be related to the fact that cortical neurons are damaged by methylmercury exposure in the form of thinning-out neuronal death. Even if a smaller number of brain neurons die, symptoms may not necessarily appear immediately, because their function can be improved by network formation called plasticity [21]. It is also known from in vitro experiments that microtubules involved in axon and dendrite formation are easily damaged at levels that do not cause nuclear or mitochondrial death [22,23], and that microtubules are reconstituted when methylmercury exposure is reduced [24,25].

These suggest that, except in the case of fulminant methylmercury toxicosis being present, this may be a complex condition involving the negative factors of methylmercury exposure and aging and the positive factor of central nervous system plasticity.

Sensory disturbances due to methylmercury exposure may improve with time [4,26,27], or they may manifest themselves slowly with prolonged exposure or disease progression [28,29,30]. This fact should be taken into account when considering the health problems caused by methylmercury toxicosis.

Our findings in Japan, discussed below, must be understood in light of the fact that they are the results of people who have been exposed to relatively high doses in large amounts or over long periods of time and decades have passed. Nevertheless, these patterns of sensory disturbance may provide insight into the sensory disturbance caused by methylmercury toxicosis.

In our study, among the somatosensory submodalities, touch and pain senses are often disturbed to about the same degree, and in severe cases both are disturbed, but in milder cases, only one of the touch or pain senses may be disturbed or the degree of disturbance may differ during the standard examination. In addition, there may be a difference in the degree of sensory disturbance between touch and pain senses, with pain disturbance being more likely to be detected than touch disturbance (Table 6).

Figure 4 shows that position sense disturbance is more likely to be detected in cases with generalized superficial sensory disturbance, but when the sensory disturbance is limited to the extremities, position sense disturbance is less likely to be detected. It is important, however, to note that any examination and quantification method also has a sensitivity limitation.

Figure S19 through Figure S24 show that both superficial touch and two-point discrimination may be impaired by cortical damage due to methylmercury toxicosis, but there are individual differences in which sense, superficial touch or two-point discrimination, is more likely to be impaired. In neurology, the disorder of two-point discrimination is often referred to as a cortical sensory deficit. This means that if two-point discrimination is impaired when superficial sensory deficits are absent or mild, the responsible lesion can be determined to be the parietal cortex. However, the reverse is not true: superficial senses may be impaired by cortical lesions, and two-point discrimination sense may not be impaired by cortical lesions.

In neurology, polyneuropathy involving peripheral nerves is considered the representative causative disorder for sensory disorders with peripheral limb predominance, and central nervous system disorders have never been introduced as a potential cause in neurology textbooks [31]. There are no known neurological disorders causing generalized sensory disturbance other than peripheral neuropathies such as hereditary sensory and autonomic neuropathy (HSAN) [32] and acute autonomic and sensory neuropathy (AASN) [33], and the differences in pathogenesis and symptoms between these peripheral neuropathies and generalized sensory disturbance caused by methylmercury toxicosis are not well understood.

The results of quantitative sensory measurements contributed to defining the foci responsible for somatosensory disturbances in methylmercury toxicosis.

Quantitative sensory measurements showed that each part of the body (lower lip, chest, upper and lower extremities) was affected to almost the same degree in the exposed group as in the control group (Figure 3, Figure 4, and Figure 6). When symptoms are due to common polyneuropathies other than HSAN or AASN, sensory disturbances in the lower extremities are more severe than in the upper extremities, and sensory disturbances in the lower lip or chest are usually absent. These findings are consistent with those of Ninomiya et al. [11,12], and suggest that the sensory disturbances are due to central rather than peripheral nerve damage.

The fact that not only residents with generalized sensory disturbance by standard touch examination, but also those with peripheral limb predominance showed similar degrees of disturbance of minimal tactile and vibration senses in the lips, chest, upper and lower extremities, and similar degrees of disturbance of two-point discrimination sense in the lower lips and bilateral index fingers, suggests that these touch disturbances are caused by central nerve damage (parietal lobe damage). At the same time, this indicates that the generalized touch disturbance and the peripheral touch disturbance in the extremities are sequential syndromes of the same cause but of different severity.

In the present study, peripheral nerve conduction velocities as well as amplitudes showed no correlation in all quantitative sensory measurements (Figure S25 through Figure S32, Figure S33 through Figure S40, Figure S41 through Figure S56, and Figure S57 through Figure S60). This further demonstrates that the sensory deficits in methylmercury-exposed cases are due to central rather than peripheral nerve damage.

Previous electrophysiologic reports of cases in Iraq [4,16,34] did not suggest peripheral neuropathy. In cases of Minamata disease in Niigata, it was reported that M waves and SNAP (sensory nerve action potential) of the ulnar nerve showed mild abnormalities [35]. There is a report that the peripheral nerve conduction velocity was delayed during the acute phase of exposure to ethylmercury and subsequently recovered [36]. Nagaki et al. reported no difference in peroneal nerve biopsy and electrophysiologic examination between eight Minamata disease patients and eight control subjects [37].

In reviewing the pathological findings, Hunter et al. reported that autopsy cases of patients with visual field constriction, ataxia, and generalized sensory disturbance showed no abnormalities in the peripheral nerves [38]. No peripheral neuropathy was found in autopsy cases of patients with early Minamata disease [39]. Eto et al. reported the presence of peripheral neuropathy on the basis of pathological findings in Minamata disease patients in a study in which not only were no controls enrolled, but the histogram of the diameter of the myelinated fibers of the peroneal nerve [40], which was shown to be abnormal in this study, was not significantly different from that of the controls [41].

Regarding Nagaki’s study, Eto pointed out that in seven of the eight Minamata disease patients, perineal biopsies were performed at a point more than 20 years after the onset of the disease, indicating that the peripheral neuropathy was cured [42]. However, Eto reported that he found no pathological findings of Minamata disease in clinically severe Minamata disease patients with Hunter–Russell syndrome who had no central lesions and peripheral nerve lesions on pathological examination [43], which means that he himself assumes the presence of central lesions for the diagnosis of Minamata disease, which is inconsistent with his claim.

In addition, when considering the diagnosis of methylmercury toxicosis, attention must be paid to the very low sensitivity of pathological findings, except in severe cases. Ikuta, who studied the pathology of Minamata disease in Niigata, described that “the pathological lesions of non-severe Minamata disease are difficult to diagnose because of the absence of specific findings such as corpuscles or inclusion bodies, and because of their ‘simplicity’ as if they were apparently similar to lesions in the aging brain” [44] and “there is almost no glial cell reaction, only neurons gradually and sporadically dropping out. It is by no means easy to immediately recognize changes such as those seen in Minamata disease, where only a 20% cell loss can eventually be detected” [44]. This means that even if no pathology can be found in the parietal cortex, the presence of neurological findings such as sensory deficits cannot be ruled out.

Thus, although the existence of mild peripheral neuropathy cannot be denied in cases of acute onset or those with massive exposure, the lesion responsible for somatosensory disturbance in cases of long-term chronic course may be the parietal cortex.

In general, most of the neurological diseases that are known to cause peripheral sensory disturbance in the extremities are polyneuropathies, such as diabetic polyneuropathy, in which peripheral nerves are affected [31], but there are several clinical differences between these and the sensory disturbance caused by methylmercury toxicosis. In polyneuropathies, such as diabetic polyneuropathy, sensory disturbances occur first in the lower extremities and are more pronounced in the lower extremities than in the upper extremities. Therefore, the presence of sensory disturbance in the upper extremities is often not essential for the diagnosis of polyneuropathy [45]. However, in the case of peripheral dominant sensory disturbances due to methylmercury toxicosis, the upper and lower extremities are affected at about the same time, and the degree and extent of upper and lower extremity involvement are similar; Figure 2, Figure 3, and Figure 5 show that the degree of upper and lower extremity deficits is similar, indicating that the upper and lower extremities are affected to about the same extent.

In addition, neurological disorders other than methylmercury toxicosis that can cause generalized sensory disturbances are very rare and include HSAN [32] and AASN [33]. In these disorders, the absence of pain sensation may be short-lived due to trauma or infection. However, in Minamata disease, although there are cases of patients with its severe form who do not feel any pain at all during trauma, the sensory disturbance in many chronic cases is milder than that caused by the peripheral neuropathy described above. A comparison of complaints and sensory levels (Figure S1 through Figure S18) illustrates this.

This is because in severe peripheral neuropathy, the tactile or painful input itself is disrupted, whereas the sensory deficits in methylmercury toxicosis are due to thinning-out of cortical neurons at various stages in the parietal lobes of the cerebral cortex, and the symptoms are milder. As a group, ADLs are more impaired in the more severely affected individuals, but there is also wide individual variation.

A weakness of this study is that the primary examining physician differed between the exposed and control groups. We believe that this problem was mitigated by the proper instruction given before the examination and by the secondary examination performed by two physicians.

In addition, the fact that the criteria for the presence or absence of complications differed between the exposed and control groups could be one of the weaknesses. However, we chose this approach because previous studies have shown that the presence or absence of complications does not have a significant effect on neurological symptoms, and because comparisons between the complicated and uncomplicated groups of the exposed subjects showed almost identical results. We believe that the much stricter criteria for complications in the exposed group than in the control group is not necessarily an exclusively positive bias.

Despite these weaknesses, this study is important from a number of perspectives. For decades, the epidemiology and sensory disturbance of Minamata disease have been poorly studied in Japan, and the diagnosis of existing patients has not been made. It can be said that conducting these studies, even if belatedly, would be helpful in elucidating the pathophysiology and diagnosis of methylmercury toxicosis and the sensory–cognitive system of the brain.

## 5. Conclusions

The peripheral limb-dominant and generalized sensory disturbances observed in chronic methylmercury toxicosis are caused by damage to the parietal cortex, and these two types of sensory disturbances are sequential phenomena resulting from the same pathophysiology.

All sensory modalities can be impaired by methylmercury exposure: superficial, deep, and cortical sensation, but the number of modalities impaired decreases as the disease becomes milder. The most common residual sensory disturbance in patients with milder disease is pain disturbance, but there are individual differences.

In chronic methylmercury toxicosis, the sensory system is more likely to be impaired than the motor system, and thus health problems are more likely to be overlooked. However, a close examination of sensory submodalities may reveal the presence of latent morbidity.

## Figures and Tables

**Table 1:** Classification of somatosensory disturbance level.

	Touch Disturbance	Pain Disturbance
Level 4	type V, IV	type V, IV
Level 3	type V, IV	type III, II, I
	type III, II, I	type V, IV
Level 2	type III	type III
Level 1	type III	type II, I
	type II, I	type III
Level 0	type II, I	type II, I

**Table 2:** Demographic characteristics of subjects in each area (*n* = 327).

		Group E	Group E + N	Exposed (Total)	Control
		(*n* = 81)	(*n* = 116)	(*n* = 197)	(*n* = 130)
Sex, *n* (%)					
	Male	26 (32.1)	54 (46.6)	80 (40.6)	51 (39.2)
	Female	55 (67.9)	62 (53.4)	117 (59.4)	79 (60.8)
Age **					
	Mean ± SD	59.9 ± 11.8	65.6 ± 9.2	63.2 ± 10.7	63.7 ± 9.3
	Range (min–max)	37–85	43–89	37–89	50–86
Smoking, *n* (%)					
	Non-smoker	46 (69.7)	74 (69.8)	120 (69.8)	102 (79.1)
	Smoker	20 (30.3)	32 (30.2)	52 (30.2)	27(20.9)
Alcohol drinking, *n* (%) *					
	Non-drinker	42 (66.7)	67 (65.7)	109 (66.1)	67 (51.9)
	Drinker	21 (33.3)	35 (34.3)	56 (33.9)	62 (48.1)
Frequency of fish intake, *n* (%) **					
	Three times a day	43 (53.8)	43 (37.4)	86 (44.1)	6 (4.8)
	Twice a day	20 (25.0)	45 (39.1)	65 (33.3)	7 (5.6)
	Once a day	15 (18.8)	21 (18.3)	36 (18.5)	26 (20.8)
	More than once a week	2 (2.5)	3 (2.6)	5 (2.6)	59 (47.2)
	Less than once a week	0 (0.0)	3 (2.6)	3 (1.5)	27 (21.6)
Occupation, *n* (%)					
	Non-Fishermen (subject) **	72 (88.9)	108 (93.1)	180 (91.4)	130 (100)
	Fishermen (subject)	9 (11.1)	8 (6.9)	17 (8.6)	0 (0.0)
	Non-Fishermen (subject’s parent) **	44 (54.3)	87 (76.3)	131 (67.2)	116 (99.1)
	Fishermen (subject’s parent)	37 (45.7)	27 (23.7)	64 (32.8)	1 (0.9)
Neurologically related complications, *n* (%) ^a^					
	Diabetes Mellitus	0 (0.0)	34 (29.3)	34 (17.3)	3 (2.3)
	Cervical Spondylosis	0 (0.0)	54 (46.6)	54 (27.4)	13 (10.0)
	Lumbar Spondylosis	0 (0.0)	15 (12.9)	15 (7.6)	0 (0.0)
	Carpal Tunnel Syndrome	0 (0.0)	37 (31.9)	37 (18.8)	0 (0.0)
	Cerebrovascular Diseases	0 (0.0)	29 25.0)	29 (14.7)	1 (0.8)
	Other Neurological Diseases	0 (0.0)	14 (12.1)	14 (7.1)	2 (1.5)
	Total	0 (0.0)	116 (100)	116 (58.9)	18 (13.8)
Family history, *n* (%) **					
	Minamata disease (−)	23 (28.4)	31 (27.0)	54 (27.6)	130 (100)
	Minamata disease (+)	58 (71.6)	84 (73.0)	142 (72.4)	0 (0.0)

* *p* < 0.05, ** *p* < 0.01 (For age, *t*-tests showed significant differences between Groups E and E + N and between the Exposed (Total) and Control groups. For the other items, the χ^2^ test was used to determine significant differences between 2 groups (Exposed (Total)/Control) and among 3 groups (E/E + C/Control).). ^a^ Criteria are different between the exposed and the control groups.

**Table 3:** Prevalence of symptoms: Answer “Always” (*n* = 327).

No	Questionnaire	Group E	Group E + N	Exposed (Total)	Control
1	Sensory numbness in both hands	43%	55%	50%	2%
2	Sensory numbness in both legs	38%	47%	44%	1%
3	Hot sensation in the hand	12%	12%	12%	0%
4	Hot sensation in the leg	17%	20%	19%	0%
5	No pain when burn or wounded	12%	19%	16%	0%
6	Difficulty in judging the adequate temperature of bath water	9%	17%	14%	0%
7	Hanging a bag with elbow or shoulder instead of holding it in your hand	32%	36%	34%	2%
8	Headache	36%	28%	32%	0%
9	Shoulder stiffness	65%	68%	67%	9%
10	Lower back pain	53%	57%	55%	6%
11	Muscle cramps	24%	33%	30%	4%
12	Disturbed vision	43%	59%	53%	3%
13	Limited peripheral vision	28%	36%	33%	1%
14	Difficulty in recognizing a thing in your sight when you continue to stare it	19%	26%	24%	0%
15	Difficulty in finding a good in the shop	39%	33%	35%	1%
16	Difficulty in hearing	27%	47%	39%	10%
17	Difficulty in understanding a word or a sentence even if you can hear it	8%	16%	13%	1%
18	Tinnitus	28%	37%	33%	6%
19	Difficulty in smelling	16%	27%	23%	1%
20	Difficulty in tasting	18%	19%	19%	0%
21	Difficulty in judging the taste of your own cooking	14%	14%	14%	1%
22	Stumbling on flat ground	4%	12%	9%	0%
23	Difficulty in wearing slippers	21%	36%	30%	0%
24	Coming off your slippers or sandals while walking	17%	29%	24%	0%
25	Difficulty in fine finger task	52%	63%	58%	0%
26	Difficulty in buttoning	14%	38%	29%	0%
27	Dropping things in the hand	14%	26%	21%	0%
28	Dropping chopsticks while eating	4%	12%	9%	0%
29	Difficulty in speaking words or sentences well	5%	18%	13%	0%
30	Hand weakness	57%	59%	58%	2%
31	Leg weakness	46%	58%	53%	2%
32	Hand tremor while moving	17%	27%	23%	2%
33	Hand tremor at rest	10%	18%	15%	1%
34	Vertigo (feeling of spinning around)	9%	10%	9%	0%
35	Swaying dizziness	8%	8%	8%	0%
36	Fainting (syncope like) dizziness	4%	4%	4%	0%
37	Dizziness when standing up	18%	13%	15%	0%
38	General fatigue	41%	39%	40%	1%
39	Difficulty in sleeping	31%	41%	37%	4%
40	Appetite loss	9%	7%	8%	0%
41	No will to do anything	23%	27%	26%	1%
42	Cannot persevere or cannot keep working	25%	38%	33%	0%
43	Feeling as if your mind has become blank or empty	3%	10%	7%	0%
44	Cannot think about anything	3%	10%	7%	0%
45	Losing your train of thought during conversation	10%	16%	14%	0%
46	Forgetfulness	32%	41%	37%	1%
47	Feeling as if you are not yourself	8%	11%	10%	0%
48	Irritation	32%	31%	31%	0%
49	Feeling sad	16%	17%	17%	0%
50	Difficulty in finding something when interrupted	22%	30%	27%	2%

Group E vs. Group E + N: *p* < 0.05 (Q12, 23, 29), *p* < 0.01 (Q16, 26.). Exposed (Total) vs. Control: *p* < 0.01 (All).

**Table 4:** Prevalence of symptoms: Answer “Always” + “Sometimes” (*n* = 327).

No	Questionnaire	Group E	Group E + N	Exposed (Total)	Control
1	Sensory numbness in both hands	90%	91%	91%	8%
2	Sensory numbness in both legs	90%	83%	86%	8%
3	Hot sensation in the hand	46%	45%	46%	0%
4	Hot sensation in the leg	56%	55%	56%	1%
5	No pain when burn or wounded	41%	46%	44%	0%
6	Difficulty in judging the adequate temperature of bath water	41%	36%	38%	1%
7	Hanging a bag with elbow or shoulder instead of holding it in your hand	68%	71%	70%	3%
8	Headache	86%	82%	83%	23%
9	Shoulder stiffness	96%	92%	94%	50%
10	Lower back pain	88%	90%	90%	51%
11	Muscle cramps	97%	88%	92%	40%
12	Disturbed vision	80%	90%	86%	19%
13	Limited peripheral vision	67%	66%	66%	8%
14	Difficulty in recognizing a thing in your sight when you continue to stare it	57%	62%	60%	2%
15	Difficulty in finding a good in the shop	78%	71%	74%	9%
16	Difficulty in hearing	62%	79%	72%	20%
17	Difficulty in understanding a word or a sentence even if you can hear it	49%	52%	51%	7%
18	Tinnitus	75%	77%	76%	17%
19	Difficulty in smelling	49%	50%	50%	6%
20	Difficulty in tasting	47%	43%	45%	2%
21	Difficulty in judging the taste of your own cooking	46%	41%	43%	2%
22	Stumbling on flat ground	64%	72%	68%	2%
23	Difficulty in wearing slippers	62%	78%	71%	2%
24	Coming off your slippers or sandals while walking	69%	78%	74%	2%
25	Difficulty in fine finger task	86%	86%	86%	9%
26	Difficulty in buttoning	53%	69%	62%	0%
27	Dropping things in the hand	76%	80%	78%	7%
28	Dropping chopsticks while eating	61%	65%	64%	1%
29	Difficulty in speaking words or sentences well	51%	63%	58%	3%
30	Hand weakness	83%	84%	84%	7%
31	Leg weakness	83%	79%	81%	5%
32	Hand tremor while moving	71%	72%	72%	6%
33	Hand tremor at rest	51%	50%	51%	1%
34	Vertigo (feeling of spinning around)	70%	61%	64%	7%
35	Swaying dizziness	57%	58%	58%	5%
36	Fainting (syncope like) dizziness	49%	43%	46%	2%
37	Dizziness when standing up	89%	78%	82%	15%
38	General fatigue	89%	82%	85%	20%
39	Difficulty in sleeping	86%	78%	81%	21%
40	Appetite loss	44%	42%	43%	3%
41	No will to do anything	85%	88%	86%	21%
42	Cannot persevere or cannot keep working	73%	74%	74%	14%
43	Feeling as if your mind has become blank or empty	56%	56%	56%	6%
44	Cannot think about anything	49%	56%	53%	2%
45	Losing your train of thought during conversation	68%	73%	71%	8%
46	Forgetfulness	96%	95%	95%	59%
47	Feeling as if you are not yourself	38%	51%	46%	0%
48	Irritation	94%	81%	86%	33%
49	Feeling sad	76%	69%	72%	19%
50	Difficulty in finding something when interrupted	81%	80%	81%	15%

Group E vs. Group E + N: *p* < 0.05: (Q11, 16, 23, 26, 48). Exposed (Total) vs. Control: *p* < 0.01 (All).

**Table 5:** Prevalence of neurological findings (*n* = 327).

	Findings	Group E	Group E + N	Exposed (Total)	Control
1	Dysarthria	17.3%	30.2%	24.9%	1.6%
2	Hearing loss	28.9%	53.7%	43.5%	7.8%
3	Visual field disturbance	27.5%	30.1%	29.0%	0.0%
4	Normal gait disturbance (distinct)	25.9%	25.4%	25.6%	0.0%
5	Normal gait disturbance (mild-distinct)	30.9%	41.2%	36.9%	0.0%
6	Tandem gait disturbance (distinct)	17.3%	34.5%	27.4%	1.6%
7	Tandem gait disturbance (mild-distinct)	66.7%	80.2%	74.6%	11.6%
8	Romberg sign	5.3%	10.3%	8.2%	0.8%
9	One-foot standing abnormality (eyes open) (distinct)	21.5%	41.4%	33.3%	1.5%
10	One-foot standing abnormality (eyes open) (mild-distinct)	62.0%	78.4%	71.8%	12.3%
11	Finger-nose test (eyes open) (distinct)	14.8%	26.7%	21.8%	0.0%
12	Finger-nose test (eyes open) (mild-distinct)	46.9%	59.5%	54.3%	0.0%
13	Adiadokokinesis (distinct)	12.5%	27.0%	21.0%	0.0%
14	Adiadokokinesis (mild-distinct)	31.3%	58.3%	47.2%	2.3%
15	Heel-knee test (distinct)	18.3%	27.3%	23.5%	0.0%
16	Heel-knee test (mild-distinct)	49.3%	55.6%	52.9%	2.3%
17	Postural hand tremor	24.3%	22.9%	23.5%	3.3%
18	Touch disturbance (four-limb peripheral)	77.8%	87.8%	83.7%	0.8%
19	Touch disturbance (oral)	23.5%	26.1%	25.0%	0.0%
20	Touch disturbance (systemic)	21.0%	16.5%	18.4%	0.0%
21	Pain disturbance (four-limb peripheral)	93.8%	95.7%	94.9%	1.5%
22	Pain disturbance (oral)	35.8%	49.1%	43.7%	0.0%
23	Pain disturbance (systemic)	35.8%	43.1%	40.1%	0.0%

Group E vs. Group E + N: *p* < 0.05: (No. 4, 5, 10, 13), *p* < 0.01: (No. 2, 9, 14): n.s.: (all others). Exposed (Total) vs. Control: *p* < 0.01 (All.).

**Table 6:** Comparison of superficial sensory disturbance.

	Pain	General (V)	General + 4 Limbs (IV)	4 Limbs (III)	1–3 Limb(s) (II)	None (I)	Total (%)
Touch							
General (V)		10	4	0	0	0	14 (7)
General + 4 Limbs (IV)		0	21	1	0	0	22 (11)
4 Limbs (III)		7	31	90	0	0	128 (65)
1–3 Limb(s) (II)		0	2	10	3	3	18 (9)
None (I)		3	1	7	0	4	15 (8)
Total (%)		20 (10)	59 (30)	108 (55)	3 (2)	7 (4)	197 (100)

**Table 7:** Age and sex of each touch disturbance category (V–I).

	Age	*n* (M/F)
General (V)	65.9 ± 9.8	14 (6/8)
General + 4 Limbs (IV)	66.8 ± 10.6	22 (12/10)
4 Limbs (III)	63.4 ± 10.0	128 (50/78)
1–3 Limb(s) (II)	57.6 ± 9.9	18 (6/12)
None (I)	61.1 ± 15.5	15 (6/9)
Control	63.7 ± 9.3	130 (51/79)

**Table 8:** *p*-values of age among each touch disturbance type by examination (* p < 0.05, ** p < 0.01).

	General (V)	General + 4 Limbs (IV)	4 Limbs (II/I)	1–3 Limb(s) (II)	None (I)
General + 4 Limbs (IV)	0.399				
4 Limbs (III)	0.182	0.080			
1–3 Limb(s) (II)	0.012 *	0.004 **	0.011 *		
None (I)	0.162	0.112	0.289	0.224	
Control	0.207	0.096	0.411	0.013 *	0.267

## Data Availability

Data are unavailable due to privacy and ethical restrictions.

## Supplementary Materials

The following supporting information can be downloaded at: https://www.mdpi.com/article/10.3390/toxics11121023/s1, Figure S1 to Figure S18: Prevalence of complaints (Q1, 2, 5, 6, 7, 8, 11, 12, 14, 19, 20, 22, 23, 24, 26, 27, 28, 32) in each sensory disturbance level; Figure S19 to Figure S24: Relation between minimal tactile sense and two-point discrimination sense (Lower lip, Right index finger, or Left index finger) (Exposed group, Control group); Figure S25 to Figure S32: Threshold of minimal tactile sense in (right, left) (index finger, toe) and (SCV, SCA) in (right, left) (median nerve, sural nerve); Figure S33 to Figure S40: Threshold of vibration sense in (right, left) (wrist, ankle) and (SCV, SCA) in (right, left) (median nerve, sural nerve); Figure S41 to Figure S56: Threshold of (upper, lower) position sense in (right, left) (index finger, toe) and (SCV, SCA) in (right, left) (median nerve, sural nerve); Figure S57 to Figure S60: Threshold of two-point discrimination sense in (right, left) index finger and (SCV, SCA) in (right, left) median nerve; Table S1: Threshold of “minimal tactile sense” in each touch disturbance type by examination; Table S2: Threshold of “vibration sense” in each touch disturbance type by examination; Table S3: Threshold of “position sense” in each touch disturbance type by examination; Table S4: Threshold of “two-point discrimination sense” in each touch disturbance type by examination; Table S5: Threshold of visual field by Goldmann’s perimeter in each touch disturbance type by examination; Table S6: Auditory acuity by audiometer in each touch disturbance type by examination; Table S7 to Table S42: Beta coefficient and 95% confidence interval in multivariate analysis on the data of Figure S25 to Figure S60.
