# Supplementary material for: Characteristics of Abnormalities in Somatosensory Submodalities Observed in Residents Exposed to Methylmercury
Source: Toxics. 2023 Dec 15;11(12):1023. doi: 10.3390/toxics11121023 (PMC10748001; doi:10.3390/toxics11121023)
Supplement: Supplementary file 1 [file toxics-11-01023-s001.zip › toxics-2756830-supplementary.pdf]

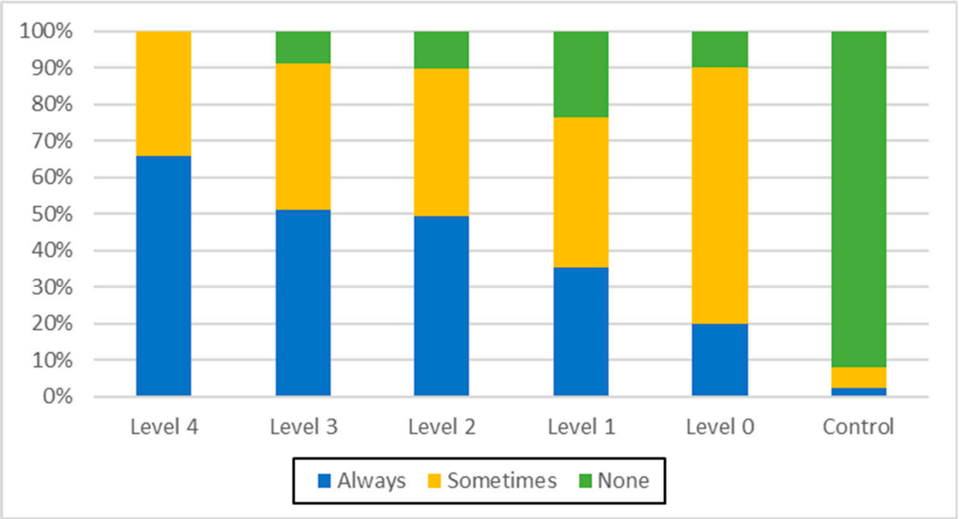

Figure S1. Prevalence of "Sensory numbness in both hands" (Q1) in each sensory disturbance level ($\chi^2(10) = 227.8$, p=0.000)

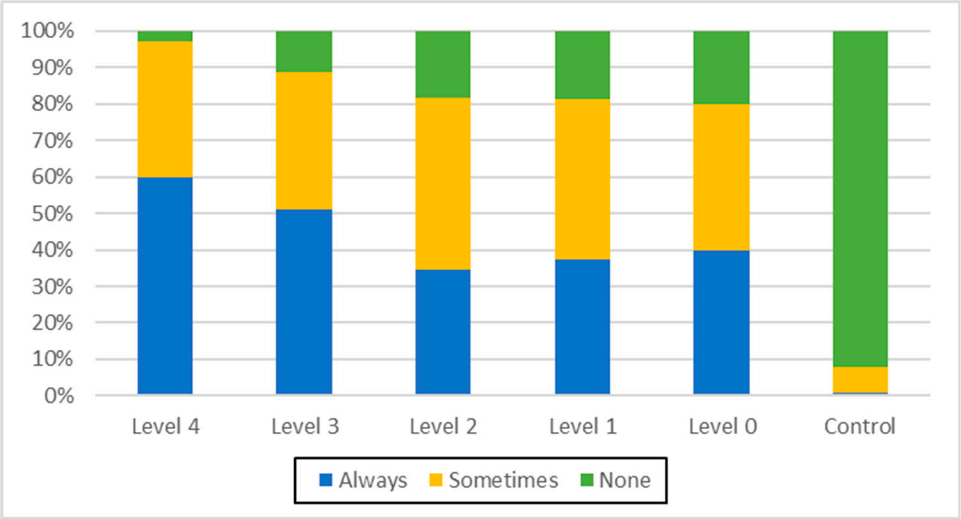

Figure S2. Prevalence of "Sensory numbness in both legs" (Q2) in each sensory disturbance level ($\chi^2(10) = 200.8$, p=0.000)

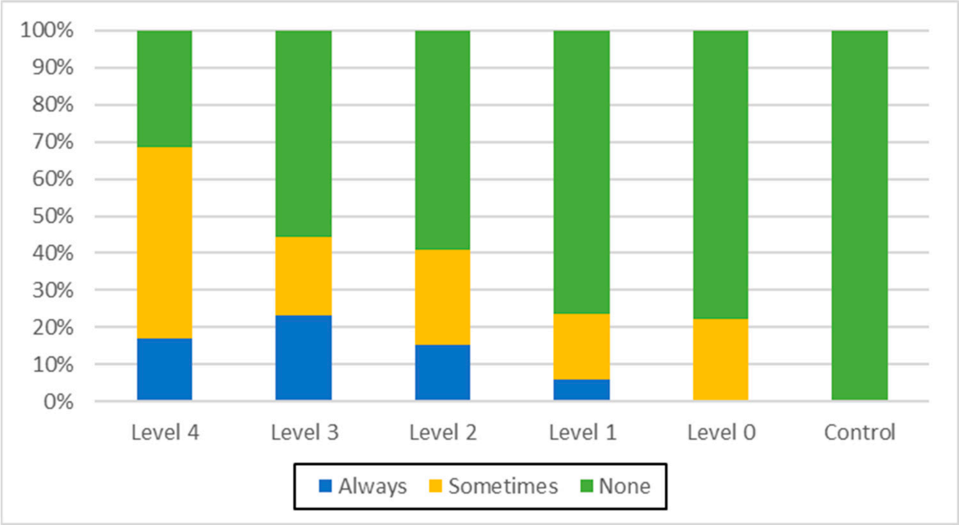

Figure S3. Prevalence of "No pain when burn or wounded" (Q5) in each sensory disturbance level ($\chi^2(10) = 101.8$, p=0.000)

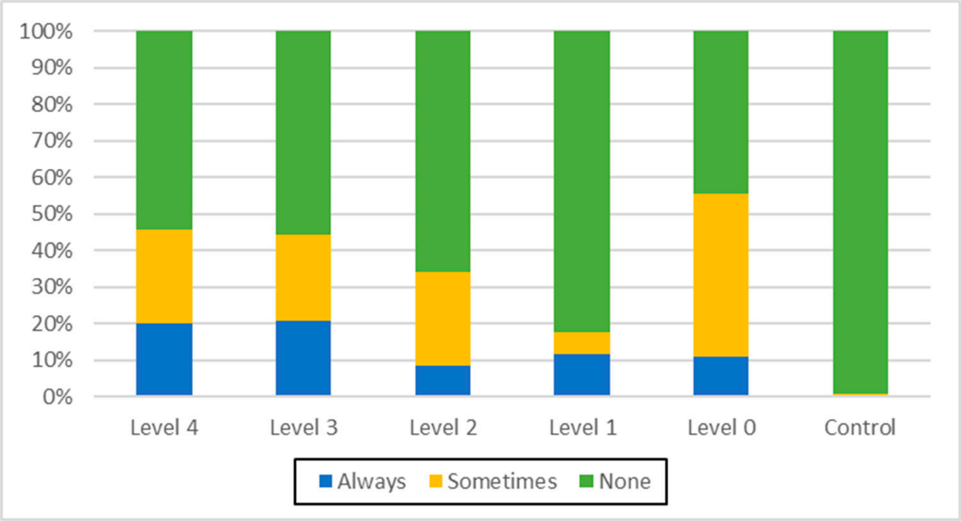

Figure S4. Prevalence of "Difficulty in judging the adequate temperature of bath water" (Q6) in each sensory disturbance level ($\chi^2(10) = 74.8$, p=0.000)

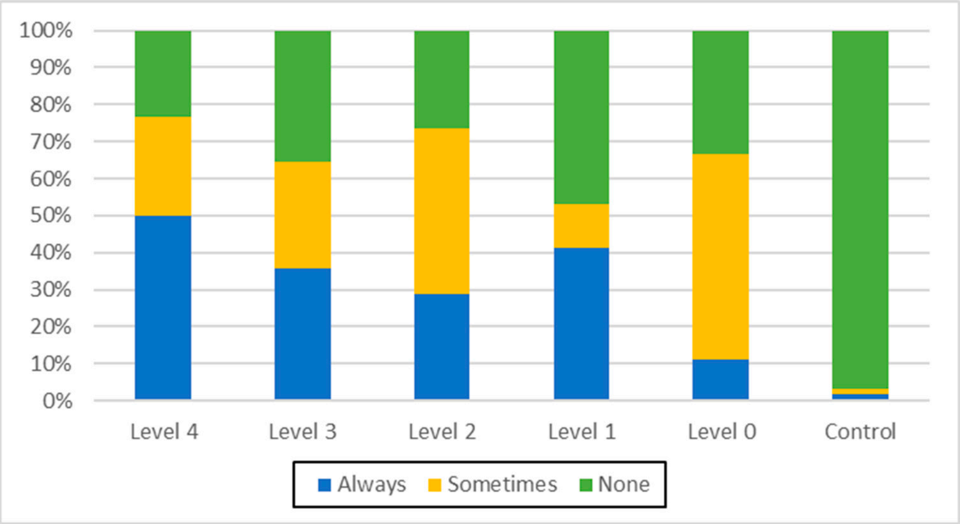

Figure S5. Prevalence of "Hanging a bag with elbow or shoulder instead of holding it in your hand" (Q7) in each sensory disturbance level ($\chi^2(10)$ =158.5, p=0.000)

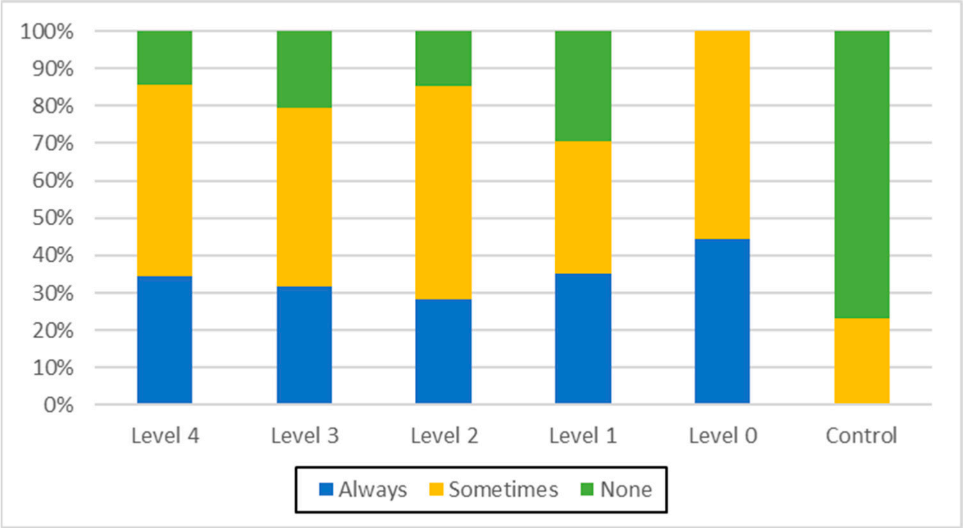

Figure S6. Prevalence of "Headache" (Q8) in each sensory disturbance level ($\chi^2(10)$ =128.2, p=0.000)

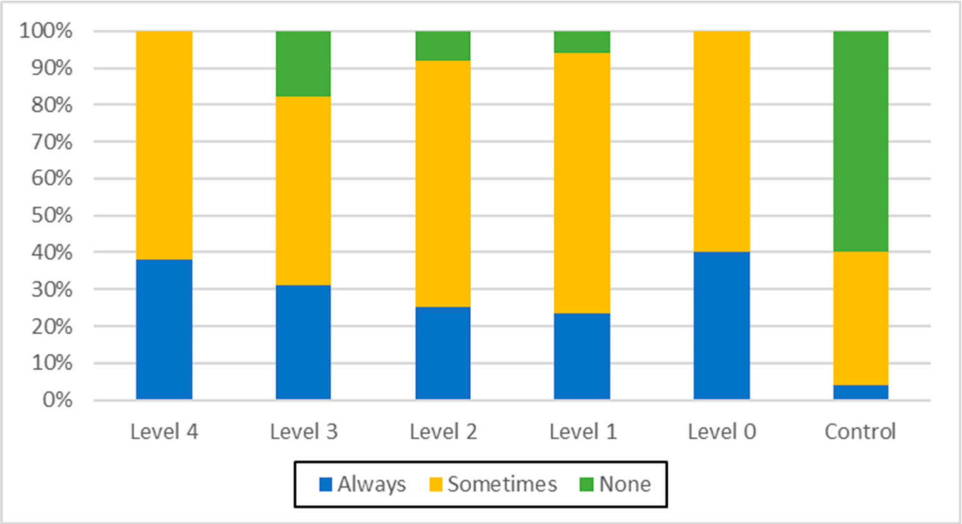

Figure S7. Prevalence of "Muscle cramps" (Q11) in each sensory disturbance level ($\chi^2(10)$ =113.5, p=0.000)

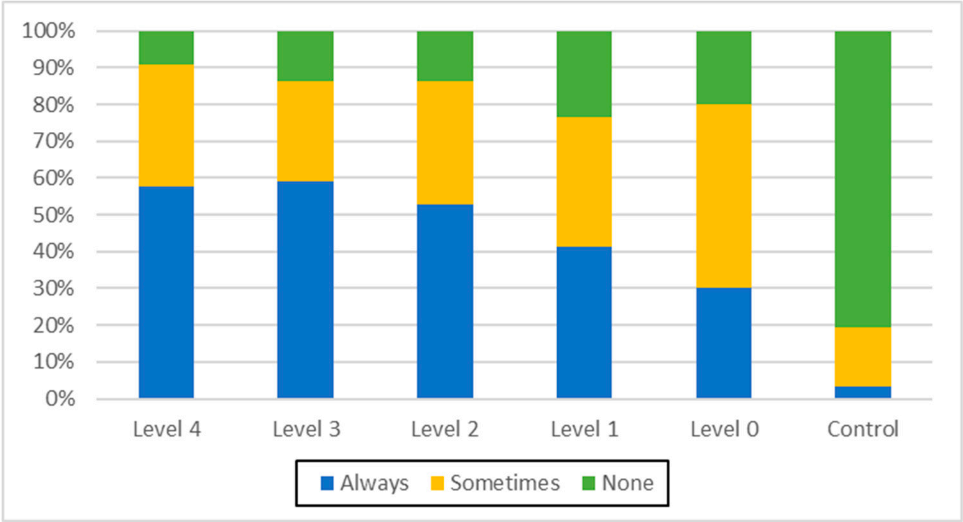

Figure S8. Prevalence of "Disturbed vision" (Q12) in each sensory disturbance level ($\chi^2(10)$ =152.7, p=0.000)

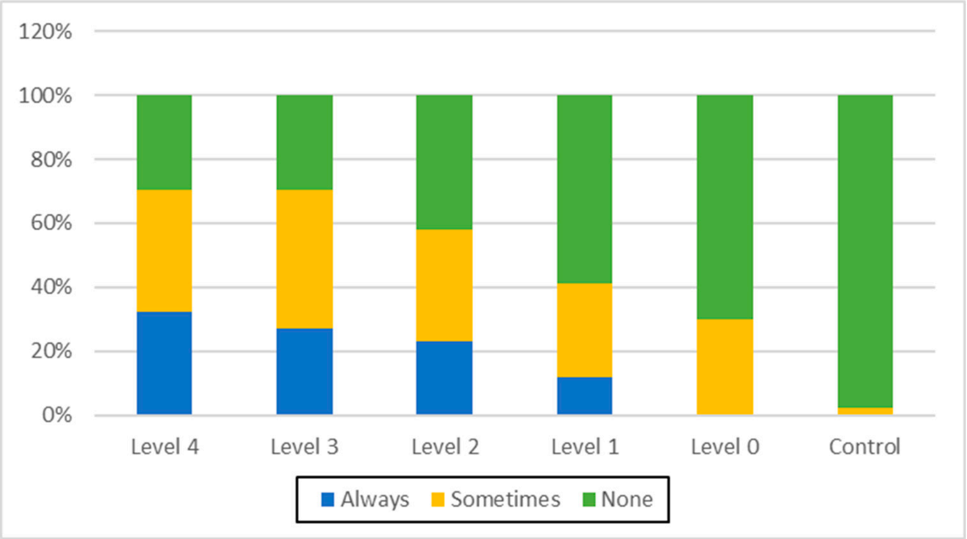

Figure S9. Prevalence of "Difficulty in recognizing a thing in your sight when you continue to stare it" (Q14) in each sensory disturbance level ($\chi^2(10) = 121.3$, $p=0.000$)

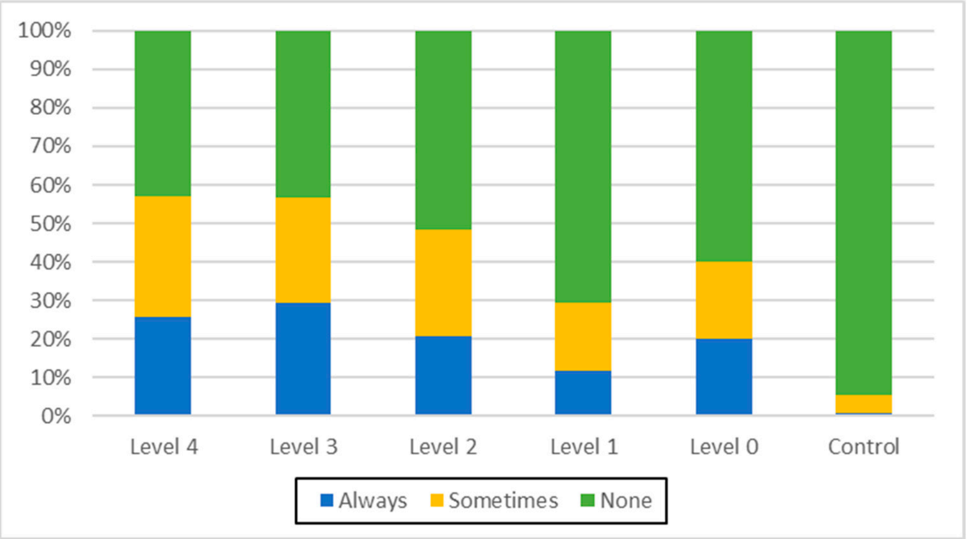

Figure S10. Prevalence of "Difficulty in smelling" (Q19) in each sensory disturbance level ($\chi^2(10) = 76.1$, $p=0.000$)

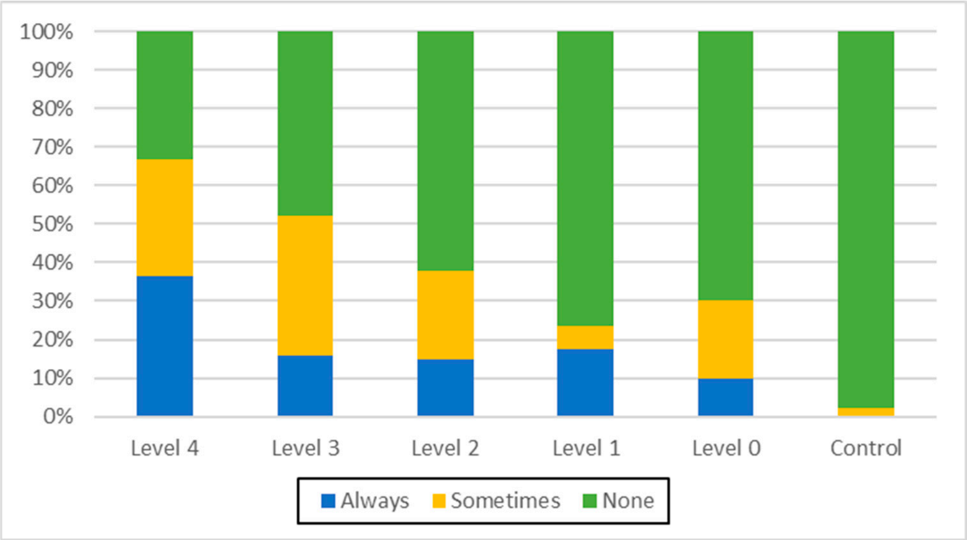

Figure S11. Prevalence of "Difficulty in tasting" (Q20) in each sensory disturbance level ($\chi^2(10) = 92.3$, $p=0.000$)

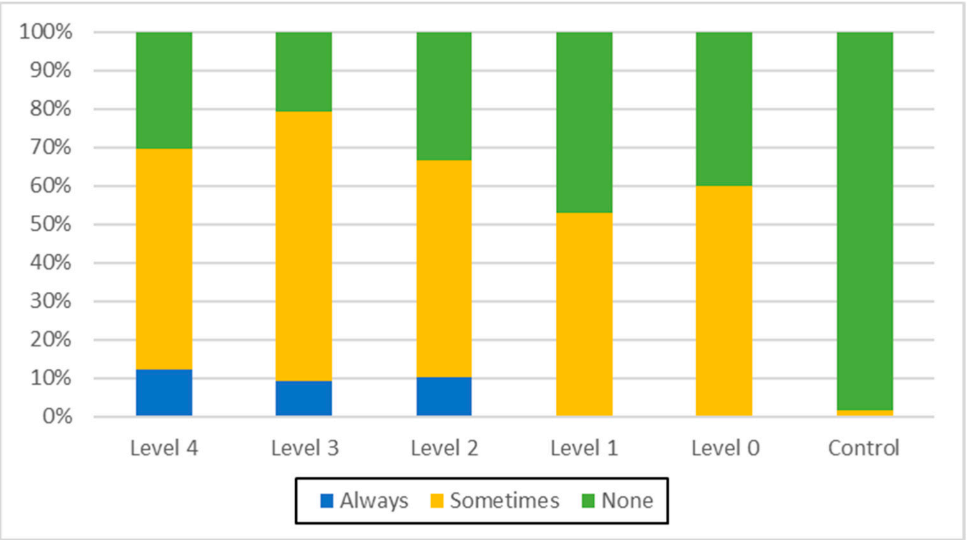

Figure S12. Prevalence of "Stumbling on flat ground" (Q22) in each sensory disturbance level ($\chi^2(10) = 149.3$, $p=0.000$)

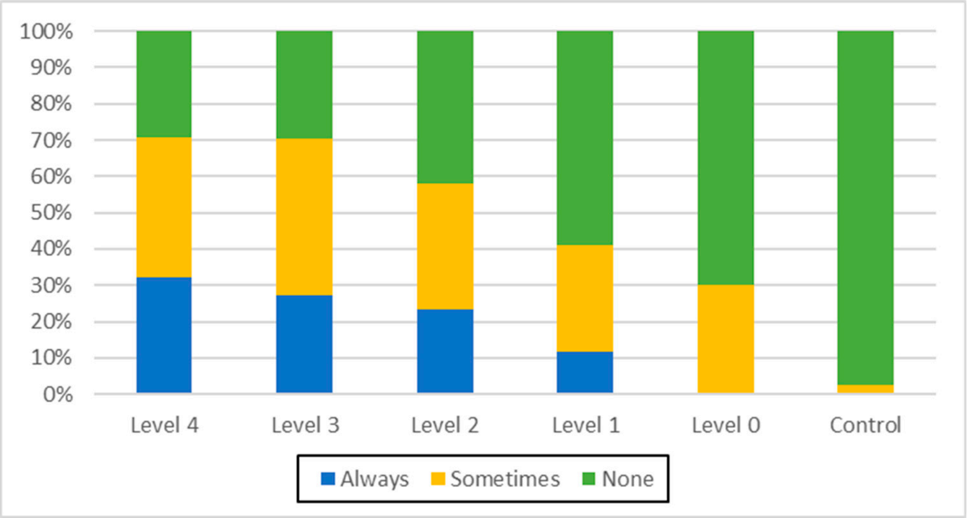

Figure S13. Prevalence of "Difficulty in wearing slippers" (Q23) in each sensory disturbance level ($\chi^2(10)$ =165.7, p=0.000)

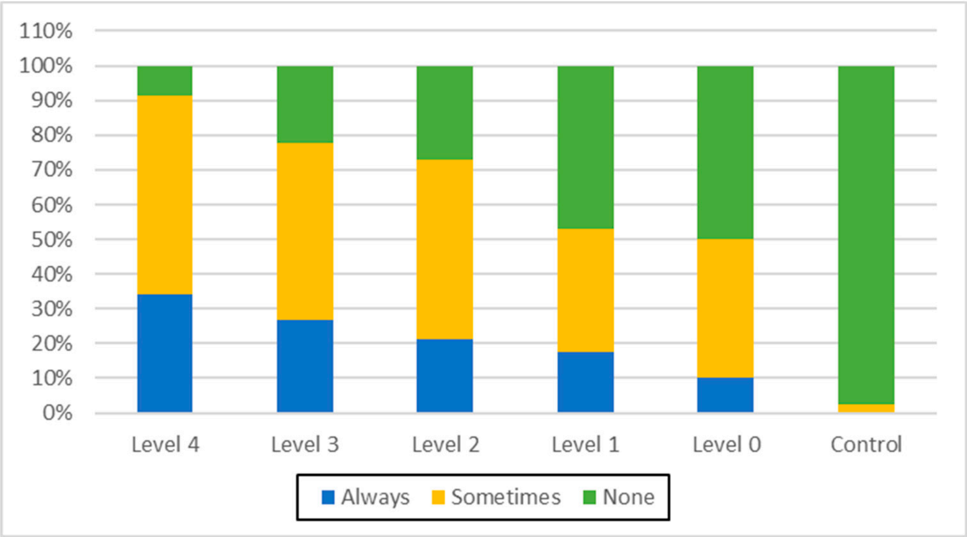

Figure S14. Prevalence of "Coming off your slippers or sandals while walking" (Q24) in each sensory disturbance level ($\chi^2(10)$ =173.0, p=0.000)

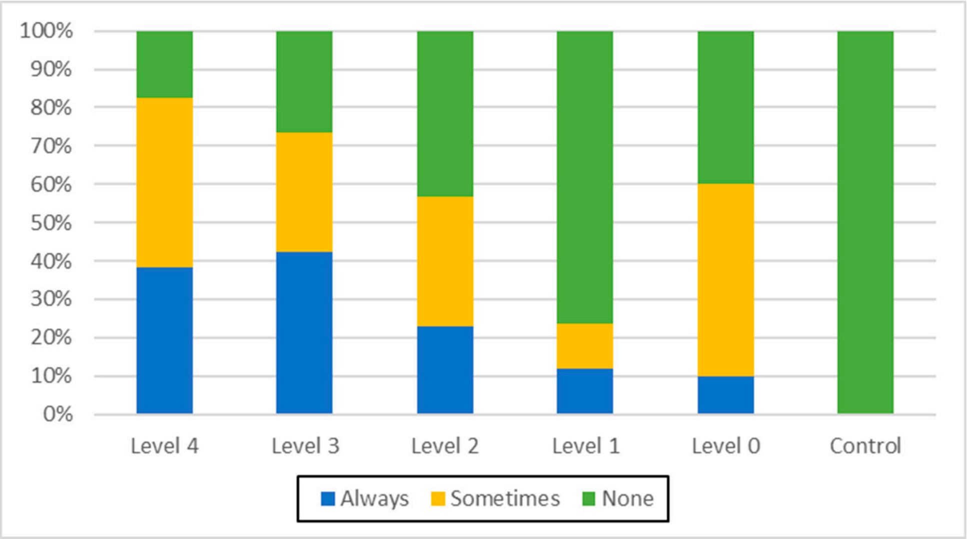

Figure S15. Prevalence of "Difficulty in buttoning" (Q26) in each sensory disturbance level ($\chi^2(10)$ =155.4, p=0.000)

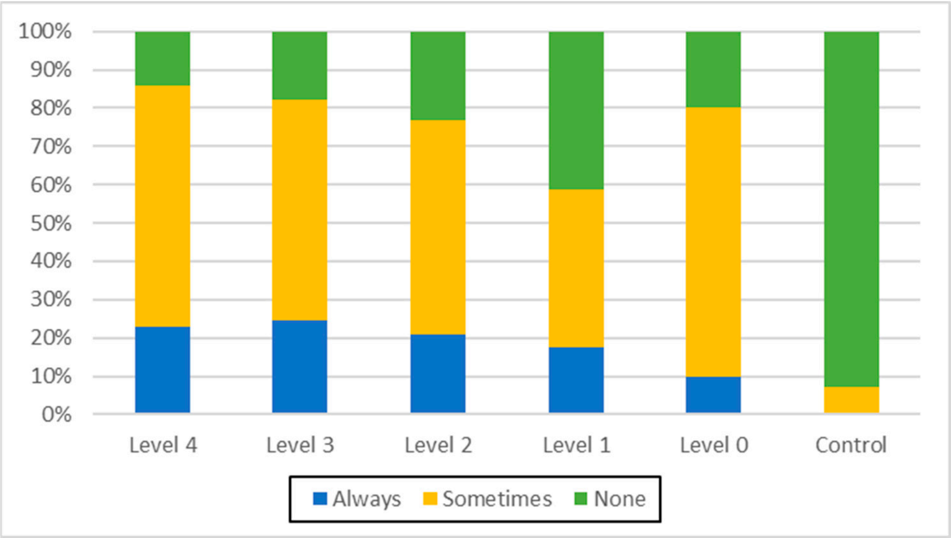

Figure S16. Prevalence of "Dropping things in the hand" (Q27) in each sensory disturbance level ($\chi^2(10)$ =162.2, p=0.000)

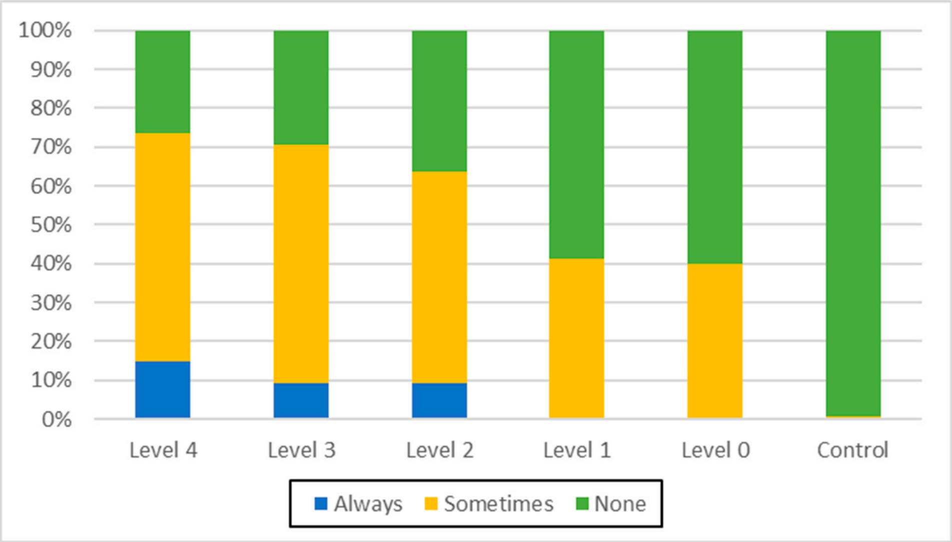

Figure S17. Prevalence of "Dropping chopsticks while eating" (Q28) in each sensory disturbance level ($\chi^2(10)$ =139.7, p=0.000)

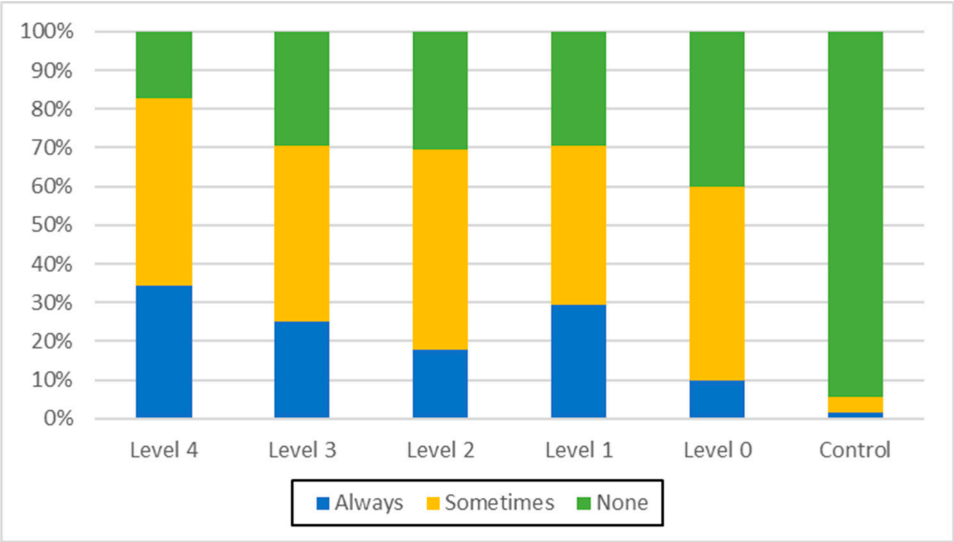

Figure S18. Prevalence of "Hand tremor while moving" (Q32) in each sensory disturbance level ($\chi^2(10)$ =142.5, p=0.000)

Table S1. Threshold of "minimal tactile sense" in each touch disturbance type by examination (Evaluator size = log([gram]) + 4)

	Lower lip	Chest	Index finger (R)	Index finger (L)	Great toe (R)	Great toe (L)
General (V)	3.66±0.79 (n=14)	4.92±0.60 (n=14)	4.96±0.68 (n=14)	4.98±0.72 (n=14)	5.98±0.58 (n=14)	5.89±0.57 (n=14)
General + 4 Limbs (IV)	3.60±0.84 (n=21)	4.82±0.45 (n=21)	4.92±0.61 (n=21)	4.93±0.76 (n=21)	5.70±0.71 (n=21)	5.71±0.69 (n=21)
4 Limbs (III)	2.64±0.55 (n=120)	4.17±0.48 (n=123)	4.23±0.52 (n=122)	4.20±0.58 (n=122)	5.01±0.67 (n=122)	5.03±0.68 (n=122)
1-3 Limb(s) (II)	2.08±0.23 (n=17)	3.73±0.50 (n=18)	3.67±0.47 (n=18)	3.67±0.34 (n=18)	4.20±0.41 (n=18)	4.18±0.46 (n=18)
None(I)	2.23±0.39 (n=13)	3.64±0.70 (n=13)	3.71±0.39 (n=14)	3.63±0.38 (n=14)	4.50±0.54 (n=14)	4.44±0.60 (n=14)
Control	2.03±0.22 (n=185)	2.82±0.58 (n=189)	3.12±0.38 (n=189)	3.04±0.35 (n=189)	3.78±0.35 (n=189)	3.81±0.40 (n=189)

Table S2. Threshold of "vibration sense" in each touch disturbance type by examination (second)

	Chest	Wrist (R)	Wrist (L)	Ankle (R)	Ankle (L)
General (V)	7.21±3.23 (n=14)	6.92±4.10 (n=14)	6.92±3.75 (n=14)	4.78±2.39 (n=14)	4.57±2.17 (n=14)
General + 4 Limbs (IV)	7.86±3.05 (n=22)	6.54±3.06 (n=22)	6.86±3.32 (n=22)	4.13±2.53 (n=22)	4.18±2.55 (n=22)
4 Limbs (III)	9.87±3.04 (n=128)	9.42±3.47 (n=128)	9.76±3.83 (n=128)	6.62±2.68 (n=128)	6.73±3.15 (n=128)
1-3 Limb(s) (II)	11.3±2.25 (n=18)	11.3±2.95 (n=18)	11.7±2.96 (n=18)	8.44±2.87 (n=18)	8.27±2.78 (n=18)
None(I)	12±2.80 (n=14)	12.1±3.84 (n=14)	12.3±4.37 (n=14)	8.42±3.45 (n=14)	8.71±3.87 (n=14)
Control	14.8±2.87 (n=196)	16.8±3.08 (n=196)	17.2±2.86 (n=196)	13.7±3.55 (n=196)	13.8±3.66 (n=196)

Table S3. Threshold of "position sense" in each touch disturbance type by examination (mm)

	Index finger (R Upper)	Index finger (R Lower)	Index finger (L Upper)	Index finger (L Lower)
General (V)	22.8±21.3 (n=14)	22.1±23.2 (n=14)	26.0±26.3 (n=14)	23.2±21.3 (n=14)
General + 4 Limbs (IV)	22.5±28.1 (n=22)	22.9±27.3 (n=22)	23.6±29.2 (n=22)	23.4±26.7 (n=22)
4 Limbs (III)	8.56±5.51 (n=128)	8.61±5.31 (n=128)	8.30±5.19 (n=128)	8.96±5.88 (n=128)
1-3 Limb(s) (II)	5.55±1.61 (n=18)	5.55±1.61 (n=18)	5.55±1.61 (n=18)	5.83±1.91 (n=18)
None(I)	6.42±2.34 (n=15)	5.71±1.81 (n=15)	6.42±2.34 (n=15)	6.07±2.12 (n=15)
Control	5±0 (n=197)	5.11±0.75 (n=197)	5±0 (n=197)	5±0 (n=197)

Table S3. (Cont.)

	Great toe (R Upper)	Great toe (R Lower)	Great toe (L Upper)	Great toe (L Lower)
General (V)	27.8±16.4 (n=14)	26.7±17.9 (n=14)	29.6±17.4 (n=14)	27.5±17.4 (n=14)

General + 4 Limbs (IV)	23.8±15.1 (n=22)	24.0±17.4 (n=22)	25.9±15.6 (n=22)	26.1±18.7 (n=22)
4 Limbs (III)	13.2±10.0 (n=128)	13.1±9.61 (n=128)	12.6±9.21 (n=128)	13.5±9.96 (n=128)
1-3 Limb(s) (II)	7.5±4.28 (n=18)	8.88±6.31 (n=18)	7.22±3.07 (n=18)	7.5±3.09 (n=18)
None(I)	8.66±7.89 (n=15)	9±6.03 (n=15)	8±7.74 (n=15)	8.33±5.87 (n=15)
Control	5.15±1.06 (n=197)	5.03±0.43 (n=197)	5.07±0.61 (n=197)	5.03±0.43 (n=197)

Table S4. Threshold of "" in each touch disturbance type by examination (degree)

	Lower lip	Index finger (R)	Index finger (L)
General (V)	31.3±12.8 (n=14)	35.8±9.16 (n=14)	36.7±8.06 (n=14)
General + 4 Limbs (IV)	17.6±13.3 (n=22)	28.2±13.7 (n=22)	29.9±12.8 (n=22)
4 Limbs (III)	9.29±10.1 (n=128)	15.4±13.7 (n=128)	16.4±14.5 (n=128)
1-3 Limb(s) (II)	4.94±3.84 (n=18)	7.5±5.45 (n=18)	8.61±8.38 (n=18)
None(I)	3.78±1.36 (n=15)	4.78±1.88 (n=15)	6.42±5.72 (n=15)
Control	2.11±0.98 (n=197)	2.82±1.20 (n=197)	3.04±1.55 (n=197)

Table S5. Threshold of visual field by Goldmann's perimeter in each touch disturbance type by examination (dB)

	Right otolateral	Right nasal	Left otolateral	Left nasal
General (V)	72.5±20.5 (n=12)	51.7±14.4 (n=12)	72.9±16.0 (n=12)	56.1±11.1 (n=12)
General + 4 Limbs (IV)	73.8±13.7 (n=20)	51.7±10.1 (n=20)	70.5±13.2 (n=20)	53.3±10.5 (n=20)
4 Limbs (III)	82.8±8.69 (n=119)	57.4±9.33 (n=118)	80.3±10.2 (n=119)	59.0±7.62 (n=119)
1-3 Limb(s) (II)	85.2±7.88 (n=18)	59.8±8.05 (n=18)	81.4±9.28 (n=17)	59.2±6.58 (n=17)
None(I)	85.3±9.63 (n=14)	59.1±9.02 (n=14)	81.3±10.8 (n=13)	61.3±6.89 (n=13)

Table S6. Auditory acuity by audiometer in each touch disturbance type by examination

	Right ear	Left ear
General (V)	33.9±12.6 (n=14)	41.7±14.9 (n=14)

General + 4 Limbs (IV)	46.2±26.2 (n=22)	45.6±26.7 (n=22)
4 Limbs (III)	30.8±15.8 (n=128)	31.3±18.1 (n=128)
1-3 Limb(s) (II)	20.5±10.8 (n=18)	21.7±11.0 (n=18)
None(I)	25.7±12.6 (n=15)	27.4±14.9 (n=15)

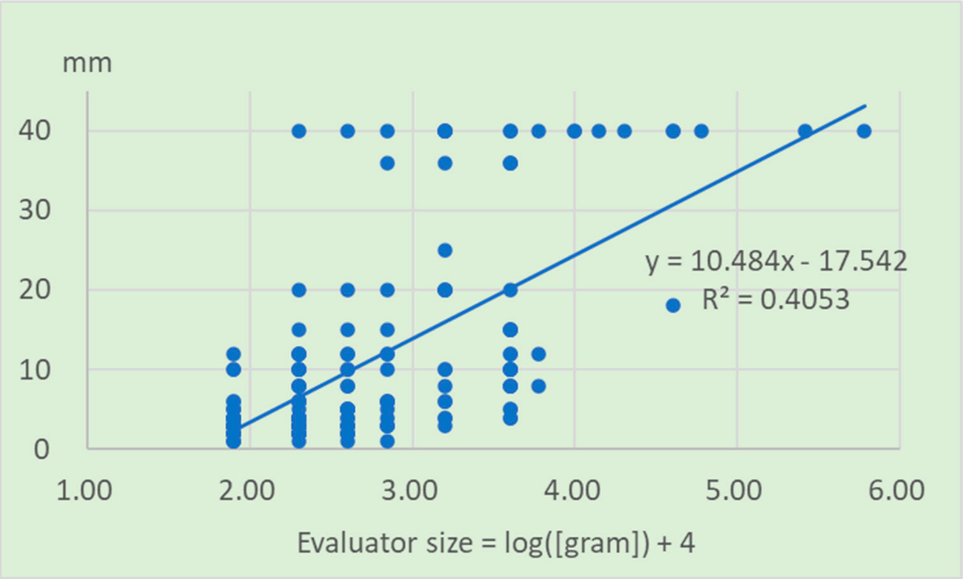

Figure S19. Relation between minimal tactile sense and two-point discrimination sense (Lower lip) - Exposed group (n=184).

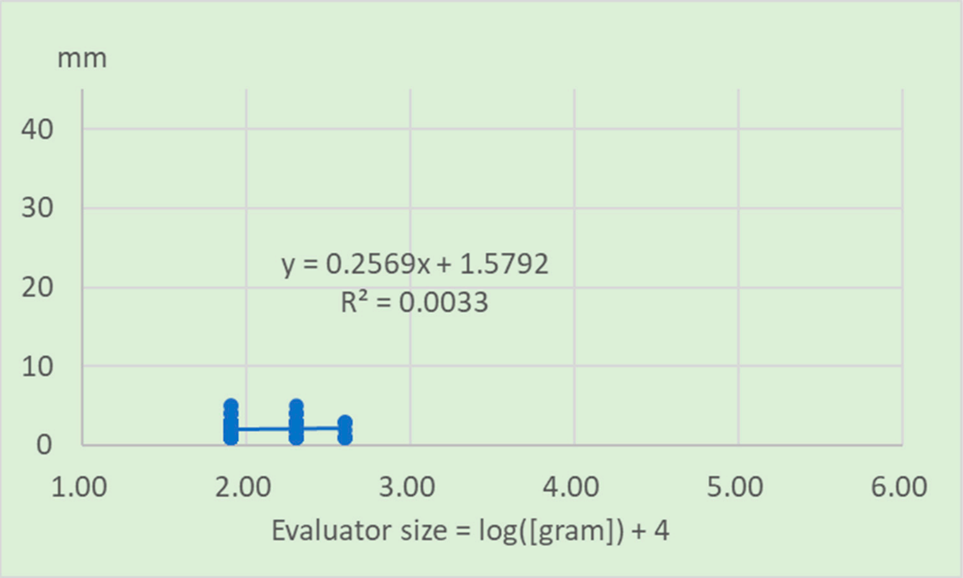

Figure S20. Relation between minimal tactile sense and two-point discrimination sense (Lower lip) - Control group (n=128).

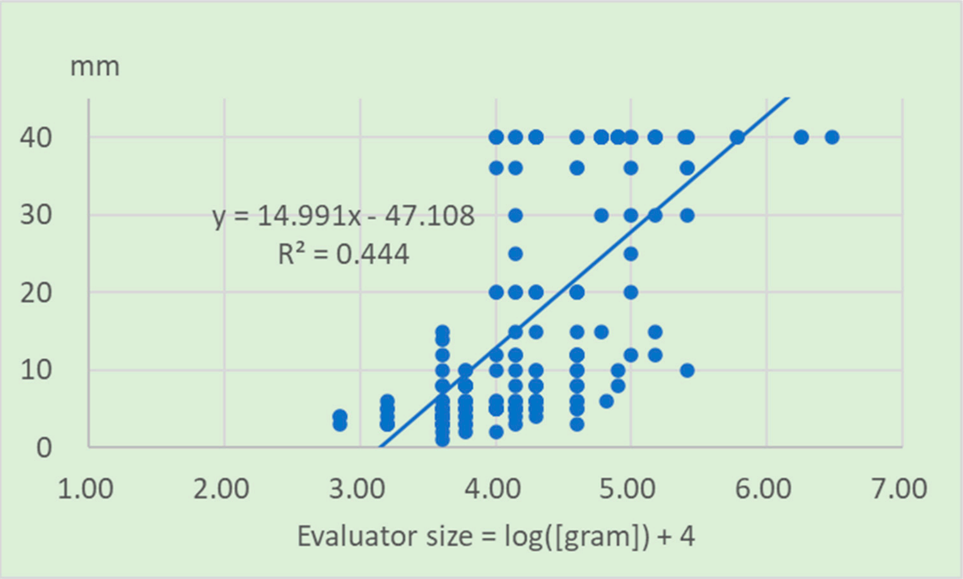

Figure S21. Relation between minimal tactile sense and two-point discrimination sense (Right index finger) - Exposed group (n=188).

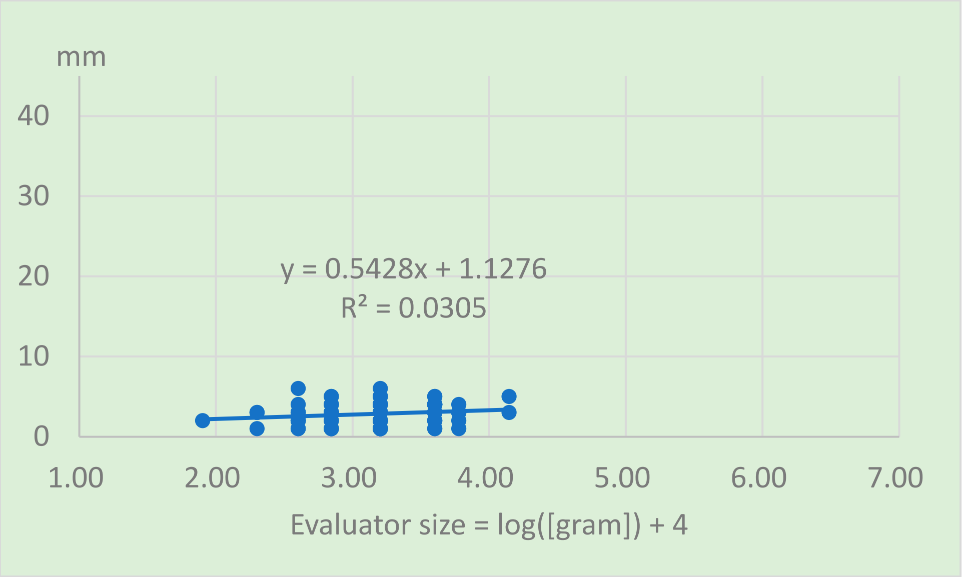

Figure S22. Relation between minimal tactile sense and two-point discrimination sense (Right index finger) - Control group (n=126).

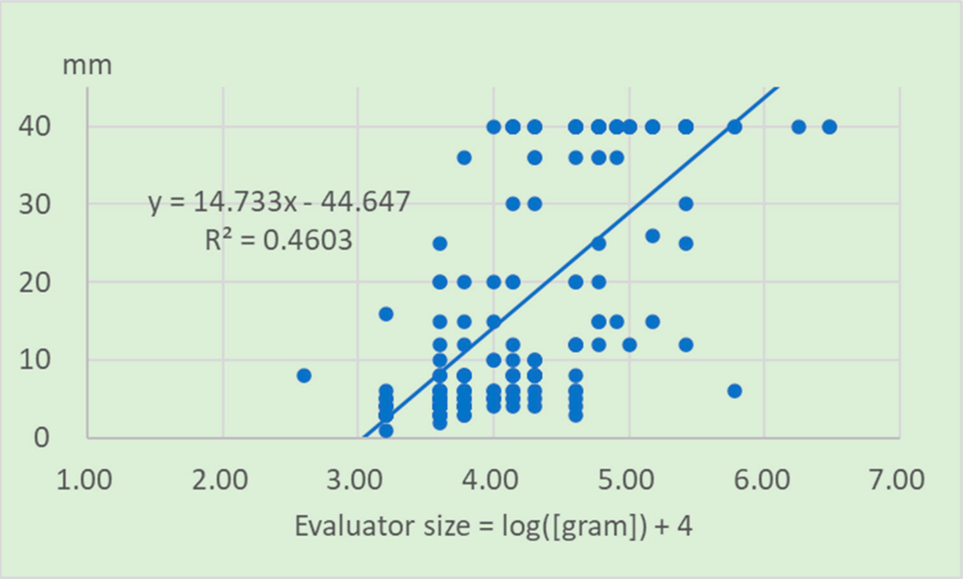

Figure S23. Relation between minimal tactile sense and two-point discrimination sense (Left index finger) - Exposed group (n=188).

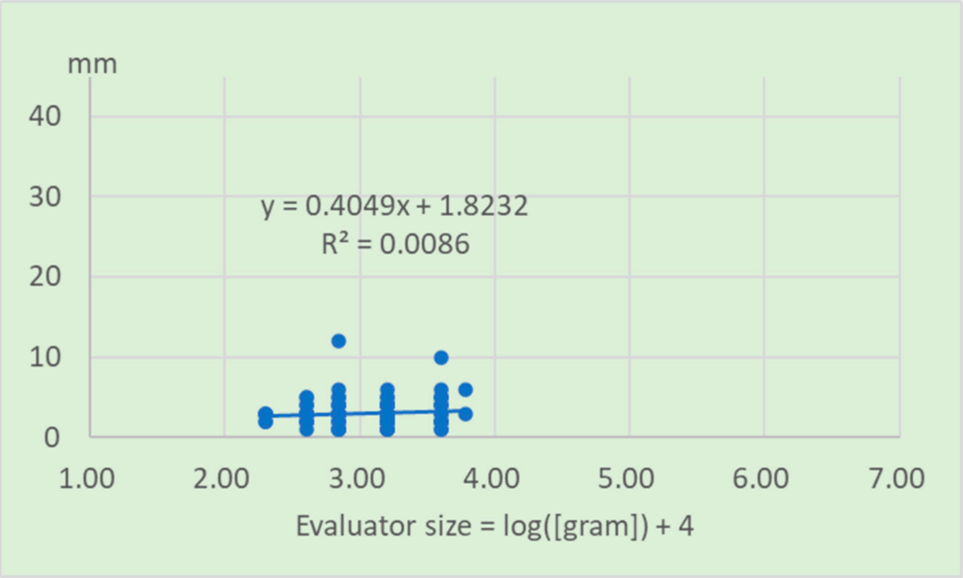

Figure S24. Relation between minimal tactile sense and two-point discrimination sense (Left index finger) - Control group (n=127).

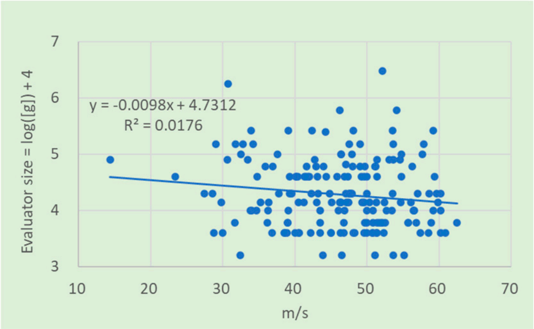

Figure S25. Threshold of minimal tactile sense in right index finger and SCV in right median nerve (n=179)

Table S7. Beta coefficient and 95% confidence interval in multivariate analysis on the data of Figure S25

	β Coefficient	95% Conf. Interval	p-value
Velocity	0.0021	(-0.009 to 0.013)	0.720
Age	0.0189	(0.010 to 0.027)	0.000
Sex	-0.1919	(-0.377 to -0.006)	0.043
Complications	0.2065	(0.002 to 0.410)	0.047

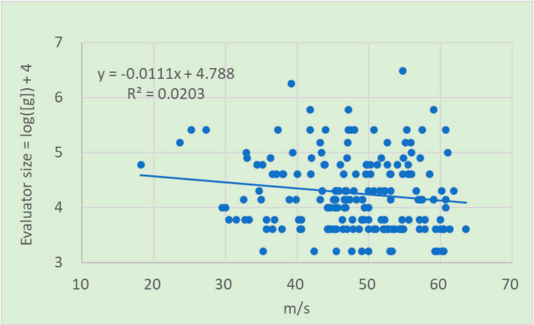

Figure S26. Threshold of minimal tactile sense in left index finger and SCV in left median nerve (n=179)

Table S8. Beta coefficient and 95% confidence interval in multivariate analysis on the data of Figure S26

	β Coefficient	95% Conf. Interval	p-value
Velocity	0.0035	(-0.009 to 0.015)	0.584
Age	0.0198	(0.010 to 0.029)	0.000
Sex	-0.2025	(-0.399 to -0.005)	0.044
Complications	0.2655	(0.041 to 0.489)	0.021

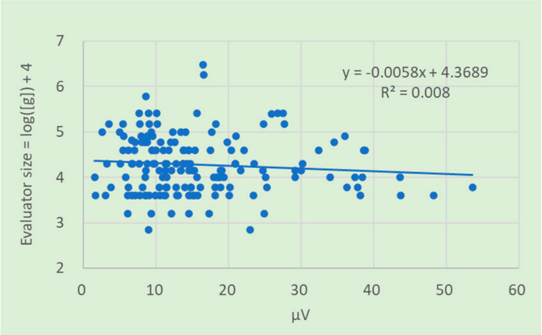

Figure S27. Threshold of minimal tactile sense in right index finger and SCA in right median nerve (n=165)

Table S9. Beta coefficient and 95% confidence interval in multivariate analysis on the data of Figure S27

	β Coefficient	95% Conf. Interval	p-value
Amplitude	0.0027	(-0.007 to 0.012)	0.609
Age	0.0204	(0.010 to 0.030)	0.000
Sex	-0.1537	(-0.349 to 0.041)	0.123
Complications	0.2265	(0.029 to 0.423)	0.025

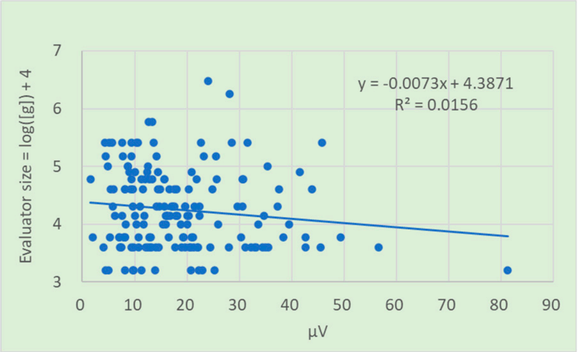

Figure S28. Threshold of minimal tactile sense in left index finger and SCA in left median nerve (n=165)

Table S10. Beta coefficient and 95% confidence interval in multivariate analysis on the data of Figure S28

	β Coefficient	95% Conf. Interval	p-value
Amplitude	0.0005	(-0.009 to 0.010)	0.991
Age	0.2104	(0.010 to 0.031)	0.000
Sex	-0.1615	(-0.376 to 0.053)	0.140
Complications	0.2593	(0.046 to 0.471)	0.017

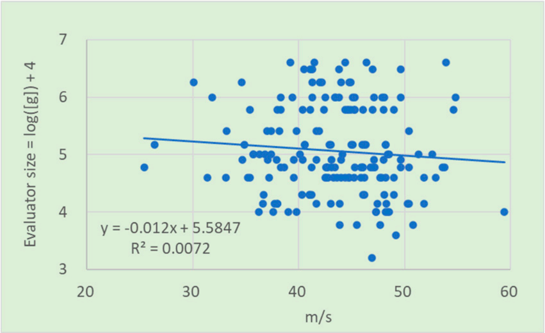

Figure S29. Threshold of minimal tactile sense in right toe and SCV in right sural nerve (n=175)

Table S11. Beta coefficient and 95% confidence interval in multivariate analysis on the data of Figure S29

	β Coefficient	95% Conf. Interval	p-value
Velocity	-0.0040	(-0.024 to 0.016)	0.710
Age	0.0215	(0.010 to 0.032)	0.000
Sex	-0.2465	(-0.476 to -0.016)	0.036
Complications	0.2844	(0.046 to 0.522)	0.019

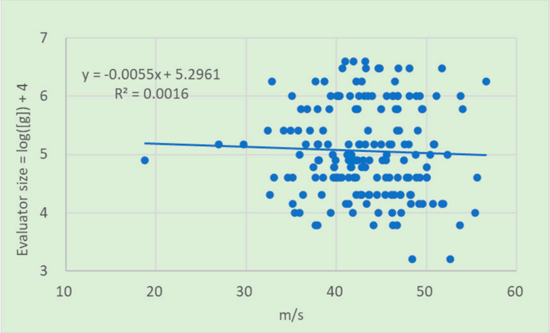

Figure S30. Threshold of minimal tactile sense in left toe and SCV in left sural nerve (n=175)

Table S12. Beta coefficient and 95% confidence interval in multivariate analysis on the data of Figure S30

	β Coefficient	95% Conf. Interval	p-value
Velocity	-0.0005	(-0.021 to 0.020)	0.961
Age	0.0197	(0.008 to 0.030)	0.001
Sex	-0.2551	(-0.490 to -0.019)	0.034
Complications	0.3490	(0.108 to 0.589)	0.005

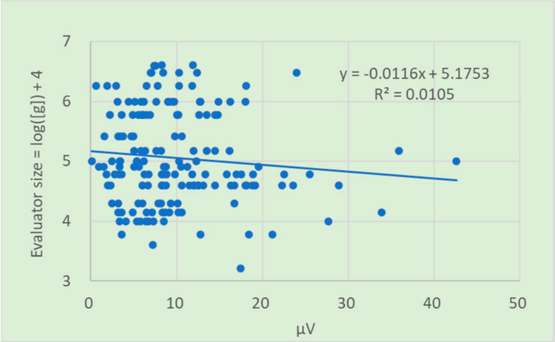

Figure S31. Threshold of minimal tactile sense in right toe and SCA in right sural nerve (n=163)

Table S13. Beta coefficient and 95% confidence interval in multivariate analysis on the data of Figure S31

	β Coefficient	95% Conf. Interval	p-value
Amplitude	0.0033	(-0.014 to 0.021)	0.721
Age	0.0232	(0.010 to 0.035)	0.000
Sex	-0.2057	(-0.446 to -0.035)	0.094
Complications	0.3203	(0.076 to 0.563)	0.010

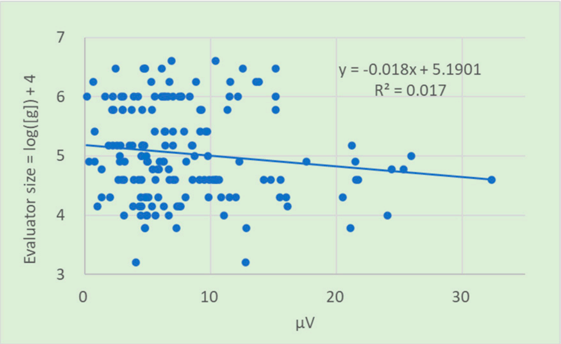

Figure S32. Threshold of minimal tactile sense in left toe and SCA in left sural nerve (n=163)

Table S14. Beta coefficient and 95% confidence interval in multivariate analysis on the data of Figure S32

	β Coefficient	95% Conf. Interval	p-value
Amplitude	-0.0047	(-0.026 to 0.016)	0.667
Age	0.0193	(0.006 to 0.031)	0.003
Sex	-0.2524	(-0.495 to -0.009)	0.042
Complications	0.3725	(0.127 to 0.617)	0.003

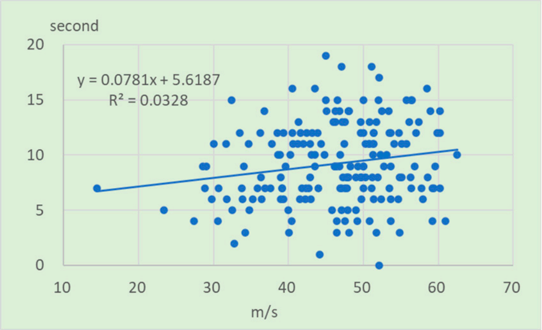

Figure S33. Threshold of vibration sense in right wrist and SCV in right median nerve (n=185)

Table S15. Beta coefficient and 95% confidence interval in multivariate analysis on the data of Figure S33

	β Coefficient	95% Conf. Interval	p-value
Velocity	0.0335	(-0.033 to 0.100)	0.327
Age	-0.0724	(-0.124 to -0.020)	0.007
Sex	0.8622	(-0.221 to 1.945)	0.118
Complications	-0.7628	(-1.944 to 0.419)	0.205

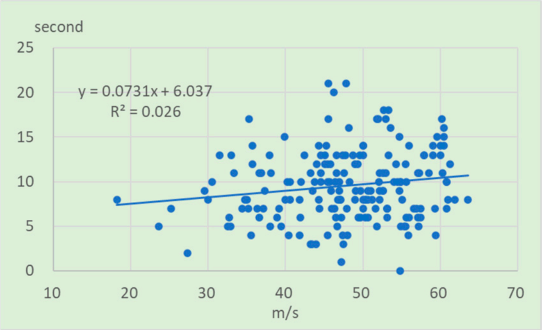

Figure S34. Threshold of vibration sense in left wrist and SCV in left median nerve (n=185)

Table S16. Beta coefficient and 95% confidence interval in multivariate analysis on the data of Figure S34

	β Coefficient	95% Conf. Interval	p-value
Velocity	0.0275	(-0.044 to 0.099)	0.455
Age	-0.0936	(-0.149 to -0.037)	0.001
Sex	0.8160	(-0.326 to 1.958)	0.160
Complications	-0.4842	(-1.772 to 0.804)	0.459

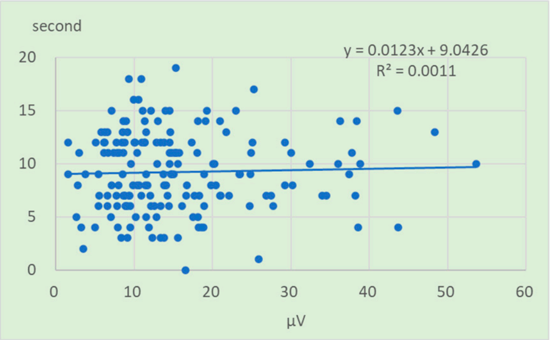

Figure S35. Threshold of vibration sense in right wrist and SCA in right median nerve (n=171)

Table S17. Beta coefficient and 95% confidence interval in multivariate analysis on the data of Figure S35

	β Coefficient	95% Conf. Interval	p-value
Amplitude	-0.0175	(-0.079 to 0.044)	0.575
Age	-0.0709	(-0.128 to -0.012)	0.017
Sex	1.0360	(-0.121 to 2.193)	0.079
Complications	-1.2193	(-2.389 to -0.049)	0.041

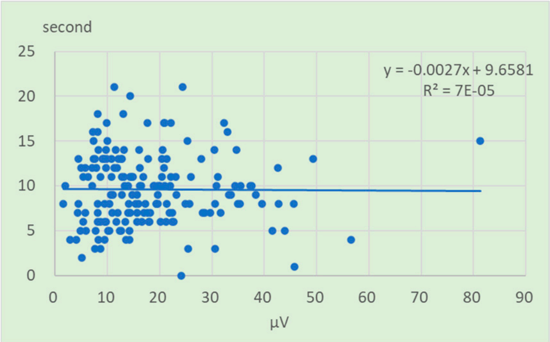

Figure S36. Threshold of vibration sense in left wrist and SCA in left median nerve (n=171)

Table S18. Beta coefficient and 95% confidence interval in multivariate analysis on the data of Figure S36

	β Coefficient	95% Conf. Interval	p-value
Amplitude	-0.0363	(-0.092 to 0.020)	0.206
Age	-0.1023	(-0.165 to -0.039)	0.002
Sex	0.7654	(-0.499 to 2.029)	0.234
Complications	-0.9630	(-2.215 to 0.289)	0.131

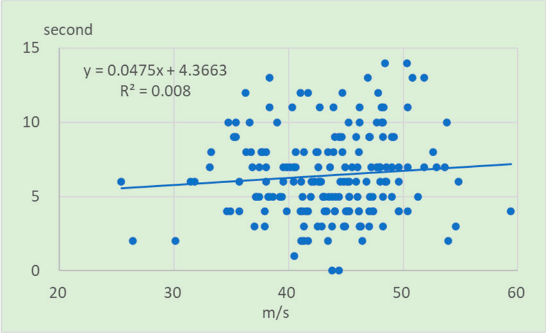

Figure S37. Threshold of vibration sense in right ankle and SCV in right sural nerve (n=179)

Table S19. Beta coefficient and 95% confidence interval in multivariate analysis on the data of Figure S37

	β Coefficient	95% Conf. Interval	p-value
Velocity	0.0257	(-0.051 to 0.102)	0.512
Age	-0.0949	(-0.135 to -0.054)	0.000
Sex	1.0058	(0.164 to 1.847)	0.019
Complications	-0.6904	(-1.555 to 0.174)	0.117

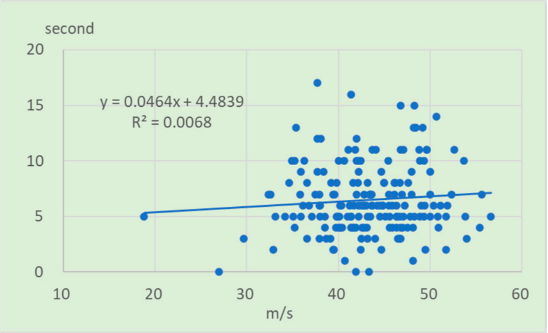

Figure S38. Threshold of vibration sense in left ankle and SCV in left sural nerve (n=180)

Table S20. Beta coefficient and 95% confidence interval in multivariate analysis on the data of Figure S38

	β Coefficient	95% Conf. Interval	p-value
Velocity	0.0355	(-0.044 to 0.115)	0.383
Age	-0.1191	(-0.162 to -0.075)	0.000
Sex	0.9899	(0.077 to 1.902)	0.034
Complications	-0.4675	(-1.394 to 0.459)	0.321

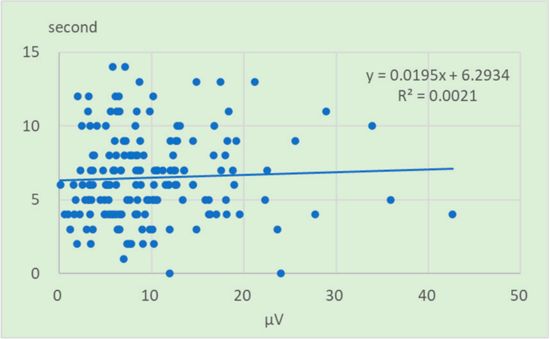

Figure S39. Threshold of vibration sense in right ankle and SCA in right sural nerve (n=167)

Table S21. Beta coefficient and 95% confidence interval in multivariate analysis on the data of Figure S39

	β Coefficient	95% Conf. Interval	p-value
Amplitude	-0.0304	(-0.096 to 0.036)	0.368
Age	-0.0965	(-0.142 to -0.050)	0.000
Sex	1.0818	(0.197 to 1.966)	0.017
Complications	-0.8948	(-1.788 to -0.000)	0.050

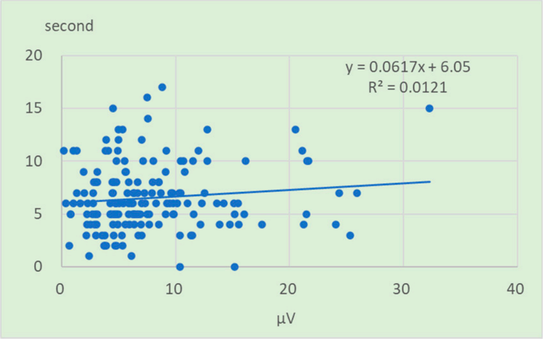

Figure S40. Threshold of vibration sense in left ankle and SCA in left sural nerve (n=167)

Table S22. Beta coefficient and 95% confidence interval in multivariate analysis on the data of Figure S40

	β Coefficient	95% Conf. Interval	p-value
Amplitude	-0.0002	(-0.086 to 0.085)	0.996
Age	-0.1129	(-0.162 to -0.062)	0.000
Sex	1.0901	(0.135 to 2.045)	0.026
Complications	-0.6522	(-1.612 to 0.307)	0.182

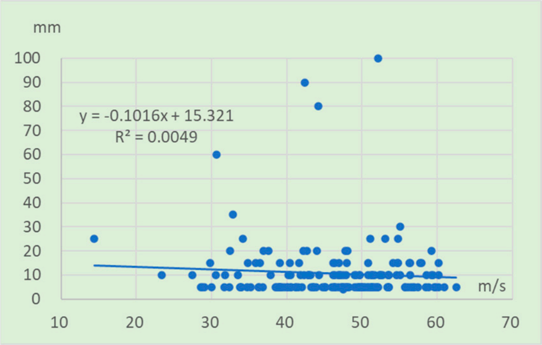

Figure S41. Threshold of upper position sense in right index finger and SCV in right median nerve (n=183)

Table S23. Beta coefficient and 95% confidence interval in multivariate analysis on the data of Figure S41

	β Coefficient	95% Conf. Interval	p-value
Velocity	0.0557	(-0.174 to 0.285)	0.663
Age	0.2141	(0.035 to 0.393)	0.019
Sex	-4.6799	(-8.392 to -0.967)	0.014
Complications	2.8550	(-1.216 to 6.926)	0.168

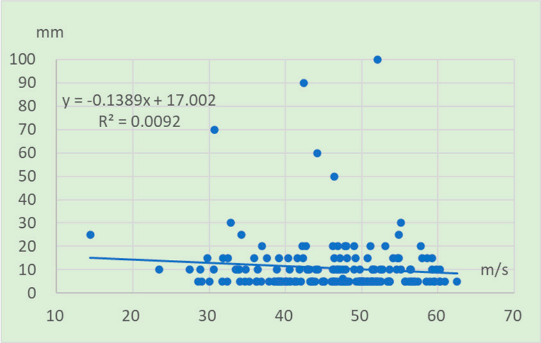

Figure S42. Threshold of lower position sense in left index finger and SCV in left median nerve (n=183)

Table S24. Beta coefficient and 95% confidence interval in multivariate analysis on the data of Figure S42

	β Coefficient	95% Conf. Interval	p-value
Velocity	0.0102	(-0.217 to 0.237)	0.929
Age	0.2408	(0.063 to 0.417)	0.008
Sex	-5.4755	(-9.148 to -1.802)	0.004
Complications	1.8874	(-2.140 to 5.915)	0.356

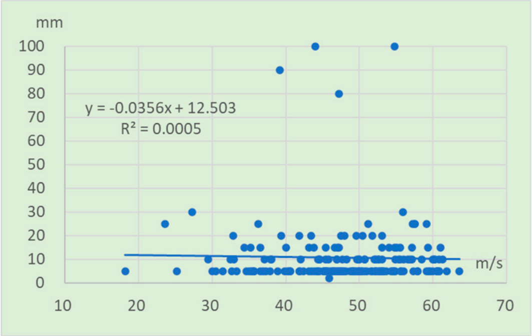

Figure S43. Threshold of upper position sense in left index finger and SCV in left median nerve (n=183)

Table S25. Beta coefficient and 95% confidence interval in multivariate analysis on the data of Figure S43

	β Coefficient	95% Conf. Interval	p-value
Velocity	0.1464	(-0.107 to 0.400)	0.258
Age	0.2706	(0.073 to 0.467)	0.007
Sex	-5.5235	(-9.543 to -1.503)	0.007
Complications	3.2714	(-1.284 to 7.827)	0.158

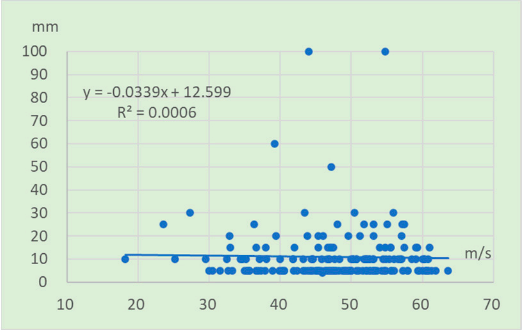

Figure S44. Threshold of lower position sense in left index finger and SCV in left median nerve (n=183)

Table S26. Beta coefficient and 95% confidence interval in multivariate analysis on the data of Figure S44

	β Coefficient	95% Conf. Interval	p-value
Velocity	0.1387	(-0.087 to 0.364)	0.227
Age	0.2645	(0.089 to 0.439)	0.003
Sex	-5.5939	(-9.163 to -2.024)	0.002
Complications	2.9815	(-1.064 to 7.027)	0.148

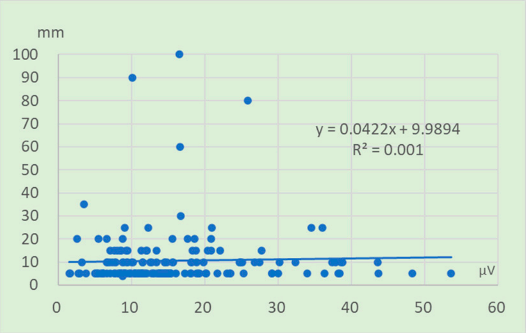

Figure S45. Threshold of upper position sense in right index finger and SCA in right median nerve (n=169)

Table S27. Beta coefficient and 95% confidence interval in multivariate analysis on the data of Figure S45

	β Coefficient	95% Conf. Interval	p-value
Amplitude	0.1260	（-0.087 to 0.339)	0.245
Age	0.2523	(0.050 to 0.454)	0.015
Sex	-4.2737	(-8.298 to -0.249)	0.038
Complications	3.1907	(-0.883 to 7.264)	0.124

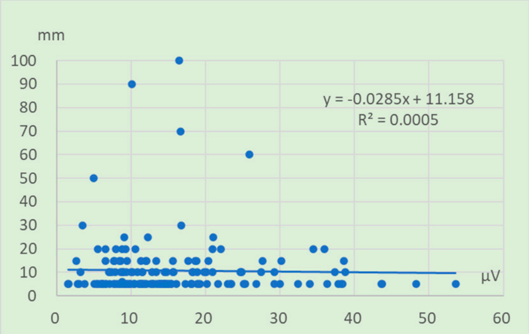

Figure S46. Threshold of lower position sense in right index finger and SCA in right median nerve (n=169)

Table S28. Beta coefficient and 95% confidence interval in multivariate analysis on the data of Figure S46

	β Coefficient	95% Conf. Interval	p-value
Amplitude	0.0312	(-0.180 to 0.242)	0.772
Age	0.2578	(0.056 to 0.458)	0.012
Sex	-5.4688	(-9.465 to -1.472)	0.008
Complications	2.2571	(-1.788 to 6.302)	0.272

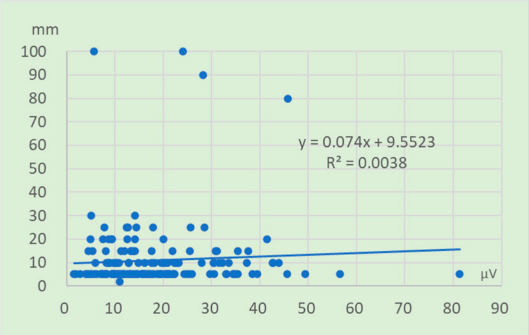

Figure S47. Threshold of upper position sense in left index finger and SCA in left median nerve (n=169)

Table S29. Beta coefficient and 95% confidence interval in multivariate analysis on the data of Figure S47

	β Coefficient	95% Conf. Interval	p-value
Amplitude	0.1572	(-0.043 to 0.357)	0.123
Age	0.3215	(0.096 to 0.546)	0.005
Sex	-4.4471	(-8.948 to 0.053)	0.053
Complications	3.1740	(-1.291 to 7.639)	0.162

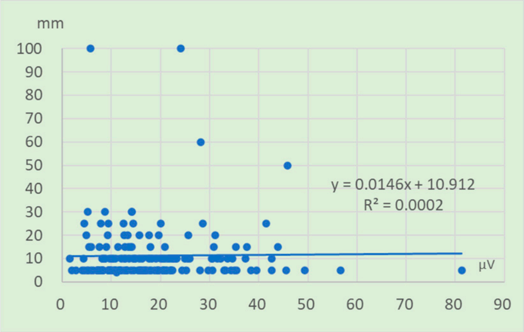

Figure S48. Threshold of lower position sense in left index finger and SCA in left median nerve (n=169)

Table S30. Beta coefficient and 95% confidence interval in multivariate analysis on the data of Figure S48

	β Coefficient	95% Conf. Interval	p-value
Amplitude	0.0752	(-0.102 to 0.253)	0.406
Age	0.2931	(0.093 to 0.492)	0.004
Sex	-5.2218	(-9.223 to -1.220)	0.011
Complications	2.7828	(-1.187 to 6.753)	0.168

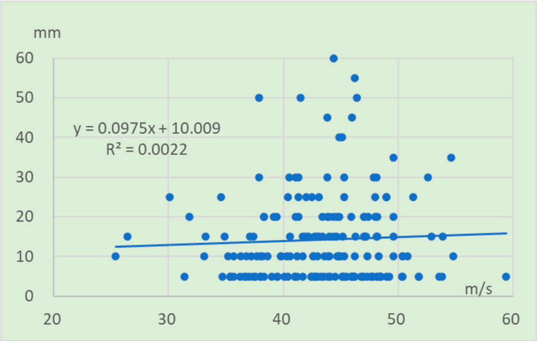

Figure S49. Threshold of upper position sense in right toe and SCV in right sural nerve (n=179)

Table S31. Beta coefficient and 95% confidence interval in multivariate analysis on the data of Figure S49

	β Coefficient	95% Conf. Interval	p-value
Amplitude	0.1461	（-0.163 to 0.455)	0.353
Age	0.3008	(0.137 to 0.464)	0.000
Sex	-3.2965	(-6.677 to 0.084)	0.056
Complications	1.3931	(-2.081 to 4.868)	0.430

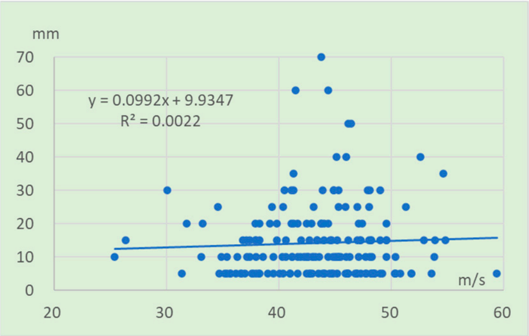

Figure S50. Threshold of lower position sense in right toe and SCV in right sural nerve (n=179)

Table S32. Beta coefficient and 95% confidence interval in multivariate analysis on the data of Figure S50

	β Coefficient	95% Conf. Interval	p-value
Amplitude	0.0910	(-0.229 to 0.411)	0.577
Age	0.2179	(0.048 to 0.387)	0.012
Sex	-4.2582	(-7.761 to -0.755)	0.017
Complications	1.0185	(-2.582 to 4.619)	0.577

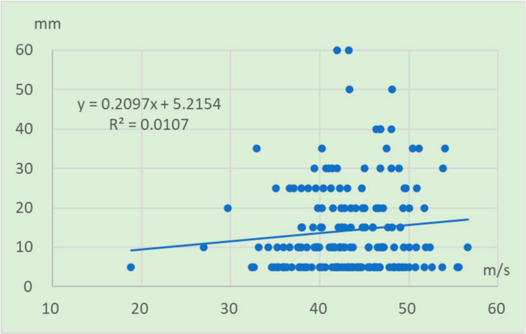

Figure S51. Threshold of upper position sense in left toe and SCV in left sural nerve (n=181)

Table S33. Beta coefficient and 95% confidence interval in multivariate analysis on the data of Figure S51

	β Coefficient	95% Conf. Interval	p-value
Amplitude	0.1873	(-0.109 to 0.484)	0.215
Age	0.3033	(0.141 to 0.464)	0.000
Sex	-4.8254	(-8.204 to -1.446)	0.005
Complications	1.4106	(-2.034 to 4.856)	0.420

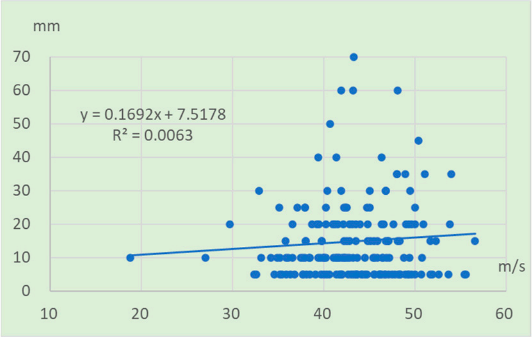

Figure S52. Threshold of lower position sense in left toe and SCV in left sural nerve (n=181)

Table S34. Beta coefficient and 95% confidence interval in multivariate analysis on the data of Figure S52

	β Coefficient	95% Conf. Interval	p-value
Amplitude	0.1254	(-0.192 to 0.443)	0.438
Age	0.2702	(0.097 to -0.442)	0.002
Sex	-5.1382	(-8.752 to -1.523)	0.006
Complications	1.0776	(-2.607 to 4.762)	0.565

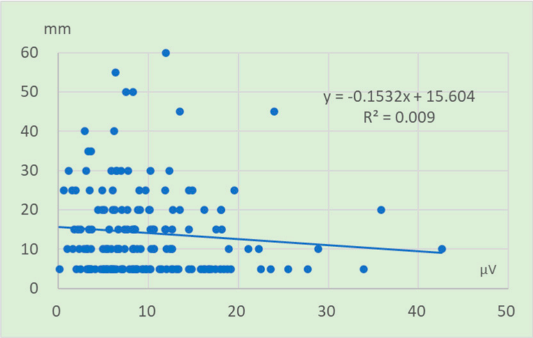

Figure S53. Threshold of upper position sense in right toe and SCA in right sural nerve (n=167)

Table S35. Beta coefficient and 95% confidence interval in multivariate analysis on the data of Figure S53

	β Coefficient	95% Conf. Interval	p-value
Amplitude	-0.0641	（-330 to 0.201)	0.635
Age	0.2310	(0.045 to 0.416)	0.015
Sex	-3.4005	(-6.939 to 0.137)	0.060
Complications	0.9923	(-2.584 to 4.569)	0.585

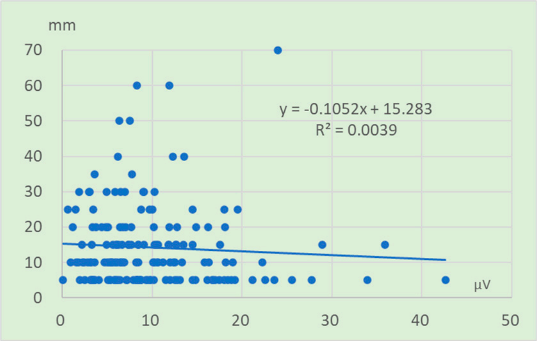

Figure S54. Threshold of lower position sense in right toe and SCA in right sural nerve (n=167)

Table S36. Beta coefficient and 95% confidence interval in multivariate analysis on the data of Figure S54

	β Coefficient	95% Conf. Interval	p-value
Amplitude	-0.4650	(-0.325 to 0.232)	0.743
Age	0.2063	(0.012 to -0.400)	0.038
Sex	-4.4357	(-8.149 to -0.721)	0.020
Complications	0.7919	(-2.962 to 4.546)	0.678

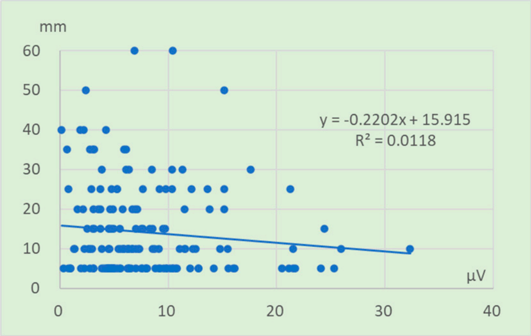

Figure S55. Threshold of upper position sense in left toe and SCA in left sural nerve (n=168)

Table S37. Beta coefficient and 95% confidence interval in multivariate analysis on the data of Figure S55

	β Coefficient	95% Conf. Interval	p-value
Amplitude	-0.1236	(-0.446 to 0.199)	0.451
Age	0.2607	(0.074 to 0.447)	0.006
Sex	-5.0349	(-8.602 to -1.467)	0.006
Complications	0.9088	(-2.692 to 4.510)	0.619

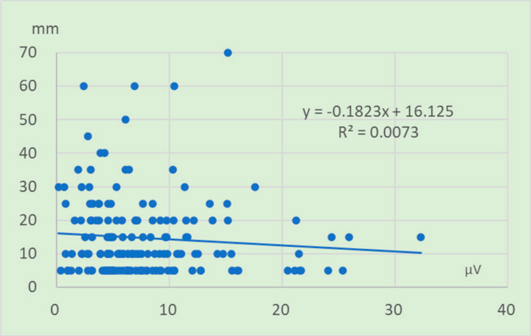

Figure S56. Threshold of lower position sense in left toe and SCA in left sural nerve (n=168)

Table S38. Beta coefficient and 95% confidence interval in multivariate analysis on the data of Figure S56

	β Coefficient	95% Conf. Interval	p-value
Amplitude	-0.1205	(-0.465 to 0.224)	0.491
Age	0.2156	(-0.016 to -0.414)	0.034
Sex	-5.2980	(-9.106 to -1.488)	0.007
Complications	0.7509	(-3.094 to 4.595)	0.586

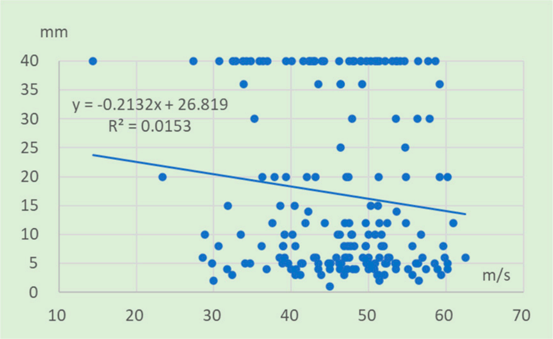

Figure S57. Threshold of two-point discrimination sense in right index finger and SCV in right median nerve (n=182)

Table S39. Beta coefficient and 95% confidence interval in multivariate analysis on the data of Figure S57

	β Coefficient	95% Conf. Interval	p-value
Amplitude	-0.0279	(-0.262 to 0.236)	0.835
Age	0.4754	(0.270 to 0.680)	0.000
Sex	-3.5775	(-7.822 to 0.667)	0.098
Complications	1.1543	(-3.495 to 5.803)	0.625

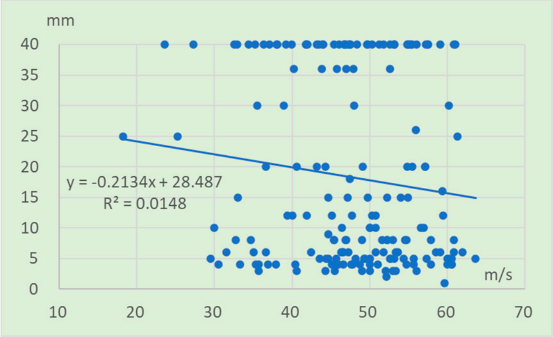

Figure S58. Threshold of two-point discrimination sense in left index finger and SCV in left median nerve (n=182)

Table S40. Beta coefficient and 95% confidence interval in multivariate analysis on the data of Figure S58

	β Coefficient	95% Conf. Interval	p-value
Amplitude	-0.0264	(-0.302 to 0.249)	0.850
Age	0.4828	(0.266 to 0.698)	0.000
Sex	-4.3877	(-8.769 to -0.006)	0.050
Complications	0.8757	(-4.072 to 5.823)	0.727

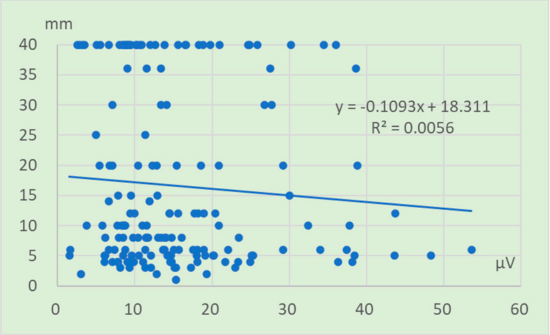

Figure S59. Threshold of two-point discrimination sense in right index finger and SCA in right median nerve (n=168)

Table S41. Beta coefficient and 95% confidence interval in multivariate analysis on the data of Figure S59

	β Coefficient	95% Conf. Interval	p-value
Amplitude	0.0083	(-0.226 to 0.243)	0.944
Age	0.4341	(0.208 to 0.659)	0.000
Sex	-3.7259	(-8.179 to 0.727)	0.100
Complications	1.0941	(-3.399 to 5.588)	0.631

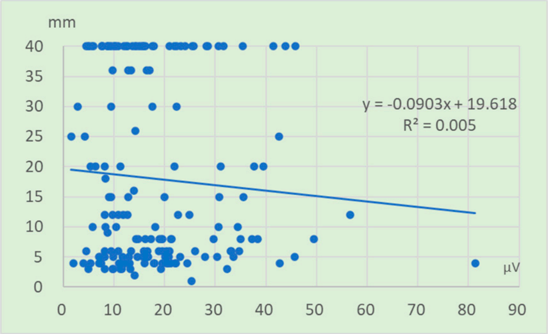

Figure S60. Threshold of two-point discrimination sense in left index finger and SCA in left median nerve (n=168)

Table S42. Beta coefficient and 95% confidence interval in multivariate analysis on the data of Figure S60

	β Coefficient	95% Conf. Interval	p-value
Amplitude	-0.0024	(-0.214 to 0.209)	0.983
Age	0.4435	(0.202 to 0.684)	0.000
Sex	-4.7731	(-9.553 to 0.007)	0.050
Complications	1.0460	(-3.679 to 5.771)	0.663
